# The Implementation of Chimeric Antigen Receptor (CAR) T-cell Therapy in Pediatric Patients: Where Did We Come From, Where Are We Now, and Where are We Going?

**DOI:** 10.46989/001c.94386

**Published:** 2024-03-13

**Authors:** Tristan Knight E, Olalekan Oluwole, Carrie Kitko

**Affiliations:** 1 Cancer and Blood Disorders Center Seattle Children’s Hospital; 2 Medicine Hematology and Oncology, Vanderbilt University Medical Center; 3 Pediatrics Vanderbilt University Medical Center

**Keywords:** Acute lymphoblastic leukemia, Axicabtagene Ciloleucel, CAR, CAR T-Cell, Leukemia, Pediatrics, Tisagenlecleucel

## Abstract

CD19-directed Chimeric Antigen Receptor (CAR) T-cell therapy has revolutionized the treatment of patients with B-cell acute lymphoblastic leukemia (B-ALL). Somewhat uniquely among oncologic clinical trials, early clinical development occurred simultaneously in both children and adults. In subsequent years however, the larger number of adult patients with relapsed/refractory (r/r) malignancies has led to accelerated development of multiple CAR T-cell products that target a variety of malignancies, resulting in six currently FDA-approved for adult patients. By comparison, only a single CAR-T cell therapy is approved by the FDA for pediatric patients: tisagenlecleucel, which is approved for patients ≤ 25 years with refractory B-cell precursor ALL, or B-cell ALL in second or later relapse. Tisagenlecleucel is also under evaluation in pediatric patients with relapsed/refractory B-cell non-Hodgkin lymphoma, but is not yet been approved for this indication. All the other FDA-approved CD19-directed CAR-T cell therapies available for adult patients (axicabtagene ciloleucel, brexucabtagene autoleucel, and lisocabtagene maraleucel) are currently under investigations among children, with preliminary results available in some cases. As the volume and complexity of data continue to grow, so too does the necessity of rapid assimilation and implementation of those data. This is particularly true when considering “atypical” situations, e.g. those arising when patients do not precisely conform to the profile of those included in pivotal clinical trials, or when alternative treatment options (e.g. hematopoietic stem cell transplantation (HSCT) or bispecific T-cell engagers (BITEs)) are also available. We have therefore developed a relevant summary of the currently available literature pertaining to the use of CD19-directed CAR-T cell therapies in pediatric patients, and sought to provide guidance for clinicians seeking additional data about specific clinical situations.

## INTRODUCTION

The advent of chimeric antigen receptor T-cell (CAR-T) therapy heralded a transformative era in pediatric oncology, offering a potentially curative therapeutic modality to young patients confronting refractory or relapsed (r/r) hematologic malignancies. Unlike the typical development course for oncologic therapeutics, which are often investigated first in adults, and only subsequently extended to children, CAR T-cell therapy was trialed simultaneously in both pediatric and adult patients. A number of key factors likely drove this. First, B-cell derived malignancies are relatively more common in children, with B-cell acute lymphoblastic leukemia (B-ALL) accounting for approximately one quarter of all pediatric cancer diagnoses^1^; they are comparatively rare in adults, accounting for less than 0.5% of new cancer diagnoses annually.^2^ Second, B-ALL is a more uniform disease in children than in adults, with both a lower mutational burden and a less heterogeneous array of mutations,^3^ simplifying study design and interpretation. Third, initial efforts to treat chronic lymphocytic leukemia (CLL) (by also targeting CD19) were somewhat disappointing,^4,5^ leading to a shift towards investigations in pediatric B-ALL (as well as towards adult diffuse large B-cell lymphoma; DLBCL). It has subsequently been found that B-ALL is particularly susceptible to CAR-T cell therapy, much more so than other CD19-positive malignancies such as CLL^6,7^ and B-lineage lymphomas.^8^ However, it should be noted that CAR-T therapies for both conditions has continued to evolve and, in the case of many B-lineage lymphomas, has become the standard of care.^9^

Although the initial clinical trials of CAR-T cell therapies focused largely on pediatric patients, the larger number of adult patients with r/r malignancies has driven the accelerated generation of adult clinical trials and expanded the indications for these therapies among adults. As of writing, only one CAR-T cell product is approved for use in children by the United States’ Food and Drug Administration (FDA) (e.g. Tisagenlecleucel) versus the six approved for use in adults. Moreover, those products are approved for use in six different diseases, and some target antigens other than CD19, versus the single indication for which Tisagenlecleucel is approved (e.g. r/r B-ALL). Finally, a large and growing disparity exists in the number of pediatric versus adult clinical trials. More than twice as many clinical trials are currently recruiting adult patients with B-ALL than are recruiting pediatric patients with B-ALL; a difference which increases sharply when examining other disease indications.^10^

A widening gap therefore exists between pediatric and adult populations, particularly regarding the opening of new clinical trials and enrollment of pediatric patients.^10^ This is also true with regard to experience and understanding of the short and long-term complications associated with CAR-T cell toxicity.^11^ The purpose of this review is therefore: (a) to outline the implementation of CAR-T cell therapy in the pediatric population, (b) to discuss both the currently available products and near-term/ongoing clinical trials, (c) to consider future indications and, (d) to explore means of improving patient outcomes. Our goal is to collate the relevant literature in these areas to provide both a resource and guidance for clinicians seeking additional information about specific clinical situations and complications arising in their pediatric patients.

## CAR T-CELL STRUCTURE, PRODUCTION, AND GENERATIONS

The first human clinical trials of CAR-T cell therapy were conducted in patients with solid tumors.^12,13^ Although some efficacy was demonstrated, both on-target toxicity and limited T-cell persistence were notable challenges. Subsequent studies investigated CAR-T cell against B-cell malignancies on the basis of (a) the efficacy of CD20-directed monoclonal antibodies, and (b) the uniquely attractive characteristics of CD19 as a target (e.g. lineage restriction, relative dispensability of cells expressing this antigen, and broad expression on B-cell-derived malignancies).^14–16^ These initial clinical trials provided the impetus for further investigations and development of CAR-T cells with improved *in-vivo* persistence. The early evolution of this treatment paradigm is outlined in greater detail in several excellent reviews.^17–19^ Similarly, the initial pioneering clinical trials have also been previously discussed.^20^

## PRODUCTION AND STRUCTURE

The production of CAR-T cells requires the collection (typically via apheresis) of peripheral blood mononuclear cells. These cells are then grown in a manner so-as to encourage the expansion and stimulation of T-cells, which are then modified to express the chimeric antigen receptor (CAR) construct. Genetic material which encodes for the desired CAR is introduced into the activated T-cells, most-often via lentiviral vectors. These viral vectors transfect the target T-cells and integrate their passenger CAR constructs into the host T-cell’s genome, thereby facilitating transgene expression. The subsequently expressed CAR construct is a modular structure comprised of (a) an extracellular antigen-binding domain (typically derived from the single-chain variable fragment (scFv) of an antibody against the target antigen), (b) a hinge domain , (c) a transmembrane domain which attaches the CAR construct to (and passes through) the cellular membrane, and (d) an intracellular signaling domain, which may or may-not include a costimulatory region.^19^

## GENERATIONS

CAR-T cell therapy has evolved through several generations, each of which is characterized by advancements in design and capabilities. These generations are best understood as iterative advances in the use of costimulatory domain(s). First-Generation CAR-T cells paired an extracellular anti-CD19 single chain variable fragment (scFv) with a single intracellular signaling domain, typically CD3 zeta (ζ), but did not possess a costimulatory domain. While the first-generation CAR T-cells demonstrate antigen-specific T-cell activation, their efficacy was impaired by limited proliferation and persistence. Second-generation CAR-T cells incorporated co-stimulatory domains alongside the CD3ζ signaling domain. The inclusion of these co-stimulatory domains, most often CD28 or 4-1BB (CD137), enhances CD3ζ signaling and facilitates improved CAR-T cell expansion and persistence and, therefore, improved activity versus the target malignancy.^21–23^ As of writing, all commercially available CAR-T cell products are second generation, e.g. they contain one costimulatory domain (CD3ζ + CD28 or 41BB).

Third-generation CAR-T cells incorporate multiple co-stimulatory domains (e.g. both CD28 and 4-1BB) in addition to the CD3ζ signaling domain, and there is evidence that they may possess superior engraftment, expansion and persistence versus second generation CAR-T cell products.^24–26^ Fourth-generation CAR-T cells utilize additional strategies, and are sometimes referred to as “TRUCKs” (T-cell Redirected for Universal Cytokine-mediated Killing / T-cell Redirected for antigen Unrestricted Cytokine-mediated Killing). They are based on second generation CAR T-cells, with the addition of a “payload” e.g. they are engineered to secrete specific cytokines (typically interleukin 12 (IL-12) or IL-18) upon antigen recognition, further promoting destruction of the target via additional synergistic mechanisms.^27^ Finally, fifth generation CAR-T cells possess a single costimulatory domain (typically CD28), but also contain a truncated, intracellular domain from the IL-2 receptor beta (β; IL-2Rβ) and a STAT3-binding motif. This combination of T-cell receptor activation via CD3ζ, stimulation via CD28, and cytokine signaling via IL-2Rβ / STAT3 provides three synergistic signals.^27^ Although relatively early in their development compared to the preceding generations, fifth-generation CAR-T cell therapies have yielded promising results in pre-clinical models, showing superior efficacy and persistence versus second-generation CAR-T products.^28^

## INITIAL CLINICAL TRIALS, AND A FOCUS ON PEDIATRIC PATIENTS

The first experience using CAR-T cell therapy to treat children with r/r B-ALL was published in 2013.^29^ This product was jointly developed by Novartis and the University of Pennsylvania, and was initially called CTL019. It was a second generation, CD19-directed CAR-T cell product utilizing CD3ζ for activation, 4-1BB for co-stimulation, and was produced via lentiviral transduction. Two children were infused (a 7- and a 10-year-old), with both initially achieving complete remission (CR). One child remains in CR, and the other suffered a CD19-negative relapse 2 months post-infusion. In both patients, cytokine-release syndrome (CRS) developed, one of whom required cytokine blockade with etanercept and tocilizumab after the failure of steroid treatment. Notably, this was the first reported use of tocilizumab for this purpose, and its use has since become standard practice.^30^

These first two patients were part of a larger, single-center phase I/IIA study of CTL019.^31^ This study enrolled a total of 30 children and adults (25 patients aged 5 to 22 years of age, and 5 patients aged 26 to 60 years of age). Of the patients treated in the pediatric cohort (n=25), 3 were in first relapse, and 22 were in second or greater relapse. Twenty patients had detectable disease at the time of infusion. The study results are not described in age cohorts, but 90% (27/30) achieved CR at the 1-month assessment. Of these patients, 26% (7/27) relapsed within 9 months of infusion – 3 from CD19-negative disease, 3 with a loss of CAR-T persistence, and 1 with progressive/refractory disease. At 6-months following infusion, the event-free survival (EFS) was 67% (95%CI, 51%-88%), and the overall survival (OS) was 78% (95%CI, 65%-95%). Detectable circulating CTL019 cells were identified in 68% of patients 6-months post-infusion. Finally, all patients experienced CRS, which was severe in 27%, requiring tocilizumab therapy.

## TISAGENLECLEUCEL

Based on the success of the previously discussed trial, a global, multi-center study was launched (CCTL019B2202 / NCT02435849 / the “ELIANA” clinical trial).^32^ The CAR T-cell product (formerly “CTL019,” now renamed “tisagenlecleucel”) was examined for efficacy in children and young adults (from 3 years of age at the time of initial eligibility screening, up to 21 at the time of initial diagnosis). Patients were required to have CD19-positive ALL, and have disease which was either refractory to induction, refractory to reinduction, in second or greater untreated relapse, relapse following hematopoietic stem cell transplantation (HSCT), or not otherwise being considered for HSCT. Patients with Ph-positive ALL were also eligible provided they received at least two prior tyrosine kinase inhibitors.

Among the 75 patients who were infused (out of 92 enrolled), the 6-month EFS and OS were 73% (95%CI, 60%-82%) and 90% (95%CI, 81%- 95%), respectively, and 1-year EFS and OS were 50% (95%CI, 35%-64%) and 76% (95%CI, 63%-86%). More than half of the patients who received tisagenlecleucel had previously undergone HSCT (61%, n=46). CRS was common following infusion and occurred in 77% of recipients (n=58). Grade 3 and Grade 4 CRS were seen in 21% (n=16) and 25% (n=19) of recipients, respectively, necessitating intensive care unit admission in 47% (n=35) of the cohort. Tocilizumab was administered to 37% (n=28) of participants. Neurotoxicity occurred in 40% (n = 30) of recipients; no grade 4 events were seen, but grade-3 neurotoxicity was observed in 13% (n=10). Infections and delayed hematological recovery were also common, occurring in 43% (n=32) and 37% (n=28), respectively. No directly attributable deaths occurred.

An updated analysis was published in early 2023 and examined 3-year outcomes of the original study cohort.^33^ In the 79 examined pediatric and young adult patients with r/r B-ALL (several of whom were enrolled after the previously reported primary analysis), the EFS was 44% (95% CI, 31%-57%), and the OS 63% (95% CI, 51% to 73%) at 3 years. Without censoring for subsequent bone marrow transplantation, 2-year and 3-year relapse-free survival (RFS) were 52.3% (95% CI, 39%- 64%) and 47.8% (95% CI, 34.4% to 60%), respectively. Additionally, no novel safety events occurred, in comparison to the primary analysis. Infections were the most common long-term (e.g. >1-year post-infusion) grade 3 or 4 adverse events, occurring in 20% of patients. B-cell aplasia at 1-and-2-years post infusion was seen in 71% (95% CI, 57.4%-81.5%) and 59% (95% CI, 43.2%-71.2%), respectively, with a median B-cell recovery at 35.3 months (95%, CI 22.9 months to not estimable).

## FDA-APPROVED CAR-T CELL THERAPIES WITH PEDIATRIC INDICATIONS

As a result of the successes discussed above,^29,31,32^ on August 30, 2017, tisagenlecleucel became the first CAR-T cell therapy to be approved by the FDA^34^ for use in the treatment of patients aged up to 25 years with refractory B-cell precursor ALL, or B-cell ALL in second or later relapse. As of writing, it remains the only FDA-approved CAR-T cell therapy for use in pediatric patients, and only for one indication (e.g. r/r B-ALL). Tisagenlecleucel has subsequently been examined in additional, non-leukemia contexts, but has not yet been approved for other indications in children. However, a phase II, single arm, multicenter study to assess the efficacy and safety of tisagenlecleucel in children and adolescents with relapsed/refractory B-cell non-Hodgkin lymphoma (NHL) completed accrual in 2021. The trial enrolled a total of 33 patients, but the results have not yet been published (NCT03610724).

## USE OF NON-FDA APPROVED CAR T-CELL THERAPIES AMONG CHILDREN

In addition to tisagenlecleucel, five other CAR T-cell therapies carry FDA-approvals for use in adult patients.^9^ Two of these, ciltacabtagene autoleucel and idecabtagene vicleucel, are second-generation CAR-T cell products which target B-cell maturation antigen (BCMA) in multiple myeloma.^35,36^ While BCMA expression has been described in pediatric B-ALL^37^ and in adults with B-ALL, Hodgkin Lymphoma (HL), and NHL,^38^ expression is highly variable and it does not appear to be a particularly promising therapeutic target in these contexts. The use of either ciltacabtagene autoleucel or idecabtagene vicleucel in children does not appear to have been described in the literature.

Of the remaining three agents (axicabtagene ciloleucel, brexucabtagene autoleucel, and lisocabtagene maraleucel), all are currently under investigations among pediatric patients.

## Axicabtagene Ciloleucel

Axicabtagene ciloleucel (KTE-C19) is a second-generation CAR T-cell therapy which utilizes a CD19-directed scFv linked to CD28/CD3 ζ costimulatory/signaling domain. It is not currently approved for any indication in pediatric patients, but is approved for use in adults with r/r large B-cell lymphoma (LBCL) (including DLBCL, primary mediastinal large B cell lymphoma (PMBCL), follicular lymphoma, and high-grade B cell lymphoma).^9,39–41^ No pediatric patients were enrolled in the cited clinical trials. As of writing, axicabtagene ciloleucel is being evaluated in one active pediatric clinical trial (NCT03642626),^42^ which is examining its use in patients aged 0-25 years with r/r B-ALL.

## Brexucabtagene Autoleucel

Brexucabtagene autoleucel (KTE-X19) is a second-generation CAR T-cell therapy product, combining a CD3ζ activation domain and a CD28 costimulatory signaling domain. It targets CD19, and is currently approved for use in adult patients (≥18 years of age) with r/r mantle cell lymphoma, or r/r B-ALL.^43,44^ Brexucabtagene autoleucel has also been evaluated in pediatric patients in the ZUMA-4 clinical trial (NCT02625480).^45^ This phase I/II multicenter study examined its use in patients aged ≤21 years of age with either r/r B-ALL or r/r B lineage NHL. Results for the r/r B-ALL cohort were recently published.^46^ The B-ALL cohort enrolled 31 patients, 24 of whom received CAR T infusion. The median age was 13.5 (range 3-20). The overall response rate (CR + CR with incomplete hematologic recovery) was 67%. For this group (n=16), all were considered MRD-negative remissions. Sixteen patients underwent subsequent HSCT (including 14 in the MRD-negative group) with a median RFS of 9.1 months (95% CI, 9.1 months – Not estimable). The 2 MRD-negative patients who did not undergo HSCT eventually died of relapsed disease. The entire cohort had a high rate of both CRS and neurotoxicity. CRS was observed in 88% of patients, but was grade 3 in 33%. Neurotoxicity was experienced in 67% of patients, with grade 3 in 4 and grade 4 in one, all of whom recovered. On the basis of the relative frequency of serious neurological events, which occurred in more than one-fifth (21%; 5/24) of infused patients, additional studies in pediatric r/r ALL were abandoned. Recruitment for ZUMA-4 is ongoing among pediatric patients with r/r B-lineage NHL. Completion of patient accrual is expected by mid 2024, but no results are as yet publicly available for the lymphoma cohort.

## Lisocabtagene Maraleucel

Lisocabtagene maraleucel (JCAR017) is a CD19-directed, second generation CAR T-cell product, with a CD3ζ activation domain and a 4-1BB costimulatory domain. It is approved for use in adult patients with r/r LBCL, including DLBCL, high-grade B-cell lymphoma, PMBCL, and follicular lymphoma.^47–49^ No pediatric patients were included in the pivotal trials noted above, and there are currently no approved pediatric indications. However, it is currently under evaluation in pediatric subjects aged ≤ 25 years with r/r B-ALL or r/r B-lineage NHL (NCT03743246), with study completion predicted in late 2024.^50^

## TISAGENLECLEUCEL IN THE REAL WORLD

Pediatric data from clinical trials of tisagenlecleucel have, as discussed previously, proven highly promising. Multiple studies have also explored its efficacy in “real-world” settings – that is, in a less highly curated patient population. Even in this group of children, the impressive results observed in the clinical trial context have largely been borne out.

One-such study included 255 pediatric and young adult patients with r/r B-ALL (median age 13 years, range 0.4 to 26 years).^51^ Eighty-six percent of patients achieved initial CR. The 6-and-12-month EFS were 69% and 52%, respectively. Six-and-12-month OS were 89% and 77%, respectively.^51^ Moreover, the frequency of CRS and neurotoxicity were also lower in the real-world analysis, occurring in 55% and 27% of children, respectively, versus 77% and 39% in the pivotal trial. Severe toxicities were also less common, with grade ≥3 CRS seen in 16% versus 41%, and grade ≥3 neurotoxicity seen in 9% versus 12%. Time -to-onset and duration of both toxicities were also essentially identical between the pivotal trial and real-world experience.

Another analysis included 200 pediatric and young adult patients with r/r B-ALL (median age 12 years, range 0 to 24 years), of whom 185 were infused.^52^ Of these 185 patients, 85% achieved CR, the 12-month OS was 72%, and the EFS 50%. Of note, the study was able to stratify children into those with undetectable, low-disease burden (e.g. < 5% bone marrow lymphoblasts / CNS1 or 2, no extramedullary disease), or high-disease burden (≥ 5% bone marrow lymphoblasts, presence of CNS or extramedullary disease). Patients with high disease burden had worse OS and EFS at 12-months (58% and 31%, respectively), versus those with low-disease burden (12-month OS and EFS 85% and 70%, respectively) or undetectable disease (12-month OS 95%, 12-month EFS 72% (p< .0001)). Toxicity also compared favorably: 63% of patients experienced CRS of any grade, and 21% experienced grade ≥ 3 CRS, while 21% experienced neurotoxicity of any grade, and 7% experienced grade ≥ 3 neurotoxicity. There was evidence that children with a greater disease burden also had a higher risk of CRS, but not neurotoxicity. Seventy-nine percent of those with high disease burden experienced CRS, versus 51% with low disease burden and 41% with undetectable disease (p<0.0001). Similarly, severe CRS was seen in 35% of those with high disease burden, versus 10% with low disease burden and 0.4% with undetectable disease (p<0.0001). Conversely, neurotoxicity did not correlate with disease burden.

A number of recent, smaller real-world studies also exist^53,54^ and present generally similar data, further validating the efficacy and safety of tisagenlecleucel in the pediatric r/r/ B-ALL setting.

## TISAGENLECLUECEL IN ‘ATYPICAL’ PEDIATRIC POPULATIONS

A number of real-world studies have also analyzed tisagenlecleucel’s efficacy in “atypical” situations, e.g. children who would not traditionally have been eligible to receive it based on standard indications, pivotal trial criteria, or otherwise would typically excluded from receiving it. These populations include children with central nervous system (CNS) relapse, infant B-ALL, and Down Syndrome (DS), to name several examples. A review article collating a large number of these analyses has recently been published,^55^ the major findings of which are highlighted below.

## CNS DISEASE

Children with lesions of the CNS, or CNS 3 status were not initially eligible for tisagenlecleucel, due to concerns that this would heighten the risk of neurotoxicity. Fortunately, subsequent experience has not borne out this concern, and patients with CNS involvement do not appear to have markedly higher rates of neurotoxicity.^56–59^ While the presence of CNS disease does not *ipso facto* indicate a higher risk of neurotoxicity, a relatively higher burden of it does appear to increase the risk of neurotoxicity,^57,59^ and as such, strategies to reduce CNS disease burden are warranted in this population. Moreover, children with CNS disease (either with or without marrow involvement) have been found to have comparable long-term OS and EFS to children with marrow disease only, and are able to achieve remission at comparable rates .^58^

## INFANT B-ALL

The feasibility of CAR T-cell therapy in infant B-ALL was not immediately clear; the aggressiveness of the disease, practical difficulties in performing leukapheresis, concerns about both T-cell function and recovery, risk of toxicities, and risk for lineage switch were all major concerns.^55^ Despite these potential issues, a number of patients with infant B-ALL have received tisagenlecleucel and several retrospective analyses have been published.^56,60,61^ The largest such study included 38 children^60^ and achieved 1-year EFS and OS of 69% and 84%, respectively, among the 28 children with infant B-ALL, despite this being a heavily pretreated population (66% of whom had previously received HSCT, and 37% of whom had previously received blinatumomab). Moreover, the incidence or severity of toxicities, as well as the rates of relapse have not been found to differ markedly from those experienced by older children.

## CHILDREN WITH DOWN SYNDROME AND B-ALL

Children with Down Syndrome (DS) and r/r B-ALL represent a uniquely vulnerable population; they suffer from particularly poor outcomes when undergoing HSCT, related both to heightened toxicities and infectious complications, and elevated risk of relapse.^62^ As such, the safe use of CAR T-cell therapy for them is a particularly attractive therapeutic approach. Based on the diminished thymic function, lessened T-cell numbers and function, greater vulnerability to cardiopulmonary toxicities, and pro-inflammatory immune microenvironment, it was initially unclear whether CAR T-cell therapy would be safe or efficacious in this group of patients. A subsequent analysis, however, including 16 patients with DS who received tisagenlecleucel, showed that CAR T-cells could be effectively manufactured, safely administered, and result in patient outcomes comparable to the non-DS population.^63^

## SPECIAL CONSIDERATIONS IN THE USE OF CAR T-CELL THERAPY FOR CHILDREN

As has been said, children are not small adults. Multiple unique considerations exist in contemplating the use of CAR-T cell therapies in pediatric patients. In the order of occurrence, these include: (A) leukapheresis, (B) lymphodepletion, (C) neurocognitive/neurodevelopmental outcomes.

## LEUKAPHERESIS

Leukapheresis in children poses both clinical and technical challenges, though these are not unique to CAR T patients. Best-practice guidelines have recently been published, the major recommendations of which are summarized below.^64^ From a technical standpoint, the sites of venous access, as well as the vascular access devices (e.g. central lines) themselves, are smaller in children than adults. Based on Poiseuille’s law, laminar flow rate is proportional to the fourth power of radius, e.g. a two-fold reduction in blood vessel radius results in a 16-fold reduction in blood flow. Pediatric patients will often require temporary placement of a dialysis grade catheter to tolerate the blood flow necessary for apheresis. The duration of collection may also be somewhat longer than in adults; in children, it is approximately 3-4 hours.^65,66^ Additionally, during leukapheresis, the extracorporeal blood volume represents a larger proportion of the patient’s total blood volume TBV.^64^ Using leuko-reduced/irradiated packed red blood cells (PRBCs) to prime the apheresis system (e.g. a “blood prime”) prior to leukapheresis should be performed if the calculated extracorporeal blood volume is expected to exceed 10-15% of the patient’s TBV.^64^ Practically, this means that children weighing less than 25 Kg should generally receive a blood prime, although it is not unreasonable to use higher weight thresholds depending on the clinical context, patient’s starting hemoglobin and apheresis system being used. In children below 10-15 Kg, pre-leukapheresis transfusion with PRBCs to a hematocrit of 40% should also be strongly considered.^64^ In children weighing at least 25 Kg and at least 8 years of age, leukapheresis collection via peripheral intravenous catheter (e.g. PIV) has been shown to be safe, effective, and to result in equivalent collection yields, without additional time requirements or adverse events (albeit following ultrasound-based pre-collection screening for adequacy of venous access).^65^ Finally, children are at a heightened risk of metabolic complications (e.g. hypocalcemia, hypomagnesemia) imposed by the use of anticoagulants/citrate exposure.^66^ The use of non-citrate anticoagulants (e.g. heparin) or a reduced ratio of anticoagulant citrate dextrose solution A (ACD-A) to whole blood is one strategy by which this risk can be reduced. Frequent monitoring for either laboratory evidence of hypocalcemia, hypomagnesemia or alkalosis, or clinical evidence of irritability, vital-sign instability or excessive crying is another strategy, with electrolyte replacement as needed.^64^

Clinically, collection of adequate numbers of functional T-lymphocytes may be difficult, particularly in children who have undergone multiple antecedent lines of therapy.^64,66^ The vast majority of children are eventually able to undergo successful leukapheresis (including those with high disease burden and who have been heavily pretreated^66,67^), but a proportion of those who are successfully collected experience manufacturing failures (1-13%.^68^ The actual timing of collection is a critical decision, particularly given the aggressive nature of the disease and multiple competing risks, including: (A) the need for sufficient numbers of adequately functional circulating T-lymphocytes, (B) risk of T-cell dysfunction induced by recent administration of chemotherapeutic agents or, (C) high marrow disease burden, and (D) concern for prolonged treatment delays given the aggressive nature of many children’s disease.^66,69,70^ Substantial washout periods exist for many commonly utilized salvage therapy regimens with most needing to be stopped 7-14 days prior to leukapheresis.^64^ Short-acting cytotoxic/antiproliferative agents, such as hydroxyurea and tyrosine kinase inhibitors, are the exception and may be stopped 3 days prior to leukapheresis.^64^ Given the inter-patient variability in children undergoing CAR T-cell therapy, it is difficult to be prescriptive about any specific time-frame, but general guidelines exist.^64^ Similarly, while the absolute lymphocyte count (ALC) needed to undergo successful leukapheresis is variable, depending on the specific protocol / product in use, generally, an ALC of at least 500 cells/L and/or a CD3 count of at least 150/L is advisable,^64,66^ and calculations of expected yield should always be performed to determine the apheresis total blood volume requirement; multiple total blood volumes and/or collection days may be necessary. Although successful collection and manufacturing has occurred in patients with lower ALCs and CD3 counts,^67,71^ this is less ideal, though reassuring for patients unable to have more robust collections.

## LYMPHODEPLETION

A number of lymphodepletion regimens are in common use in children undergoing CAR T-cell therapy.^72^ Briefly, the key goals of lymphodepletion are to (A) to reduce competition for cytokines which promote lymphocyte survival, and (B) to remove immunosuppressive T-regulatory cells.^72^ Its use improves the survival and proliferation of CAR T-cells and carries a corresponding improvement in response rate and, in particular, fludarabine-containing regimens were found to improve outcomes in adult patients.^73^ In children, fludarabine dosing is based upon body surface area (BSA), but this results in inter-patient variability in plasma concentrations^74^ and, in HSCT, fludarabine exposure has been associated with variation in EFS and transplant-related mortality.^75^ These observations inspired investigations into optimization of fludarabine exposure in children undergoing fludarabine-based lymphodepletion prior to CAR T-cell therapy.^76,77^ One study, which included 152 children and young adult patients receiving tisagenlecleucel for r/r B-ALL (median age 12.5 years, range, <1 to 26 years) identified an optimal fludarabine exposure area-under-the-curve (AUC) ≥13.8 mg × h/L.^77^ Those who received doses of less than 13.8 mg × h/L had a 2.5-times higher risk of relapse (hazard ratio (HR) 2.45; 95% CI, 1.34-4.48; p=0.005) and double the risk of relapse in case of recovery of the B-cell aplasia (HR 1.96; 95% CI, 1.19-3.23; p=0.01), when compared to patients who had fludarabine exposures of ≥13.8 mg × h/L. A separate study examined 26 children and young adult patients (median age 14.4 years, range, 4.0-24.5 years) who received tisagenlecleucel for r/r B-ALL.^76^ An AUC of ≥14 mg x h/L was found to predict superior outcomes; patients with exposures below this threshold had median leukemia-free survival of 1.8 months (versus 12.9 months, p<0.001), and a 100% chance of CD19-positive relapse within 1 year of infusion (versus 27.4%, p=0.0001). Persistence of B-cell aplasia at 6 months was also markedly lower in the under-exposed cohort (77.3% versus 37.3%; p=0.009). The authors of both studies therefore advocated for the use of personalized, pharmacokinetic-based dosing of fludarabine, although the specific target AUC has not been definitively established, and prospective studies are needed.

## LATE EFFECTS / NEUROCOGNITIVE OUTCOMES

By virtue of the relatively long time-frame for which HSCT has been an accepted treatment for r/r B-ALL, its late effects are relatively well characterized; a number of excellent reviews have been published on the topic^78–80^ and extensive guidelines exist for long-term follow up and late effects’ surveillance.^81^ Conversely, CAR T-cell therapy is a relatively novel modality and its long-term late effects, including neurocognitive outcomes, are not as well characterized. Irrespective of whether neurotoxicity occurs in the acute setting, pediatric patients experience a distinct deleterious effect on quality-of-life measures (including cognitive function) immediately following CAR T-cell therapy; however, this effect appears to diminish over a period of several months post-infusion.^82^ In an adult cohort of 40 patients who received CAR T-cell therapy for a variety of indications and were followed for 5 years post-infusion, 19 (47.5%) reported at least one ongoing cognitive or clinically significant psychiatric late effect.^83^ Younger age and pre-existing anxiety or depression were significantly predictive of worse mental health outcomes following therapy. A separate study followed 117 adult patients undergoing CAR T-cell therapy for NHL for 1-year post-infusion.^84^ From a baseline of 33% of patients experiencing neurocognitive dysfunction at time of infusion, an increase to 48% was seen at day 90, with an improvement to 35% at day 360; these rates were reported to be similar to other adult oncologic populations. Other notable findings were a significant association between the number of prior lines of therapy and worsened neurocognitive outcomes, and no association between presence/absence of acute neurotoxicity and subsequent cognitive dysfunction.

Similar studies in children are lacking but will be important to perform to characterize developmental outcomes. One key challenge, however, is isolating the specific effects of CAR T-cell therapy from that of other prior chemotherapy and/or radiation therapy which, as has been discussed, also carry neurocognitive / developmental toxicities. Ongoing monitoring and follow-up is therefore recommended for children undergoing CAR T-cell therapy, and guidelines from the Children’s Oncology Group (COG) have recently been published.^85^ In particular, these recommendations highlight the need to focus specifically on (A) children who have experienced CRS and neurotoxicity, (B) those under age 6 years at time of treatment, (C) children who have also undergone HSCT.

Despite the relative unknowns in this area, based on the well-described and relatively frequent late-effects of HSCT, particularly in young children, it seems likely that CAR T-cell therapy is less-toxic and better-tolerated than the alternatives.

## ALTERNATIVES TO CAR T-CELL THERAPY: BISPECIFIC T-CELL ENGAGERS

Apart from CAR T-cell therapy, children with r/r ALL have two other options for treatment: bispecific T-cell engagers (BITEs) or HSCT . Combinations involving more than one of these options have also been utilized.

Briefly, BITEs are specifically engineered constructs which simultaneously target two antigens: one on a malignant cell and one on a cytotoxic T cell, thereby facilitating cross-linkage and cytotoxic lysis of the target cell.^86^ A comprehensive review of the BITE oncologic treatment landscape is available.^86^ Within the context of pediatric r/r B-ALL, blinatumomab is the sole FDA-approved BITE therapy and is available for use in children with CD19-positive r/r B-ALL (including Ph-positive B-ALL). This indication overlaps with that of tisagenlecleucel, and while a head-to-head comparison would be valuable, no such study currently exists. A directly comparative study of tisagenlecleucel versus blinatumomab in adults was planned, but subsequently withdrawn and no results are available.^87^

However, an indirect comparison of tisagenlecleucel and blinatumomab has been published,.^88^ That investigation compared the results of the pivotal ELIANA (tisagenlecleucel; n = 79)^32,33^ and MT103-205 (blinatumomab; n = 70)^89,90^ trials. The authors compared rates of CR and OS between the studies, utilizing multiple statistical approaches to control for inter-study variability and differences in patient characteristics. They found that the use of tisagenlecleucel was associated with a significantly higher rate of CR versus treatment with blinatumomab, irrespective of the statistical approach utilized (odds ratios: 6.71-9.76), as well as a superior OS versus blinatumomab (as evidenced by a 68-74% lower hazard of death; (hazard ratios: 0.26-0.32)). Although it could be argued that this analysis was based on the best possible available results (and that subsequent studies/real-world experiences have shown somewhat inferior outcomes), both pivotal trials included in the analysis were likely biased in the same direction, and the general comparative approach still has merit.

It is also critical to note that as physicians become more familiar with both treatments, the two therapies are not used in the same clinical scenarios despite both being approved for r/r B ALL. Blinatumomab is being incorporated in multi-agent intensive chemotherapy plans, both for initial high risk patients as well as relapsed patients. It has also been used as a “bridge” to HSCT and unlike CAR T-cell therapy, is not generally considered to be a curative, single agent treatment for children with r/r B-ALL. The Children’s Oncology Group (COG) study “AALL1331” (91–93) included 255 patients with “low-risk” relapses (defined as marrow relapses ≥ 36 months or isolated extramedullary relapse ≥ 18 months, with MRD <0.1% following intensive re-induction chemotherapy), and did find evidence that blinatumomab plus intensive chemotherapy may be sufficiently curative and was superior to chemotherapy alone.^91^ A group of 208 children with intermediate (marrow relapses ≥ 36 months or isolated marrow relapse ≥ 18 months with MRD ≥ 0.1% following intensive re-induction chemotherapy) or high risk relapses (marrow relapses < 36 months or isolated marrow relapse < 18 months), received standard reinduction followed by two cycles of blinatumomab and consolidative HSCT (92). This treatment strategy was quite effective in this context, and the study was closed early for efficacy; the 2-year DFS and OS were 41% and 59% in those who did not receive blinatumomab, versus 59% (p=0.05) and 79% (p=0.005) for those who did, respectively. Moreover, 45% of children who did not receive blinatumomab were able to proceed to transplant, versus the 73% of those who received it (p<0.0001). A potential contributing factor to more patients on the blinatumomab arm being able to proceed to transplant is the improved toxicity profile of consolidation blinatumomab compared to that of traditional consolidation chemotherapy prior to transplant (91–93). In particular, infection-related complications were minimized, irrespective of relapse risk status. Among children with high-or-intermediate risk relapses receiving blinatumomab versus standard chemotherapy, 15% versus 65% experienced infections, 5% versus 58% experienced febrile neutropenia, 2% versus 27% experienced sepsis, and 1% versus 28% had mucositis (92,93). In children with low-risk relapses, the figures were similar, with febrile neutropenia seen in 3% versus 48% (p < 0.001), infections seen in 5% versus 51% (p < 0.001), sepsis seen in 0% versus 11% (p < .001), and mucositis present in 1% versus 7% (p = 0.018).^91^

A pair of “point / counterpoint” articles has also recently been published^92,93^ and provides an excellent overview of the relative merits of each therapy. Although not pediatric-specific, the major clinical trials of both CD19-directed CAR T-cells and CD19-directed BITEs are summarized.^92^ In essence, the arguments in favor of BITEs can be distilled down to: (A) a marginally superior safety profile, (B) the ability to titrate / escalate doses, (C) their “off-the-shelf” availability e.g. more rapid time to implementation and lack of need for manufacture, and^4^ a relatively lower reliance on the T-cell compartment for efficacy (while still necessary, T-cells are not required to be harvested for manufacture).^93^ Conversely, the arguments in favor of CAR T-cell therapies are: (A) greater overall efficacy, (B) superior trafficking to extramedullary disease, (C) the ability to address higher disease burdens.^92^ Blinatumomab appears to be less efficacious with higher disease burden, particularly above 50% bone marrow involvement.^94^ Both papers highlight the possibility of a combinatorial approach utilizing both therapies, but admittedly, studies are lacking. However, until additional data exist, this option should be utilized with caution, given the evidence that blinatumomab may diminish CD19 antigen density, which may, in turn, affect CAR T-cell effectiveness.^95–97^

## ALTERNATIVES TO CAR T-CELL THERAPY: HEMATOPOETIC STEM CELL TRANSPLANTATION

Apart from BITEs, HSCT is the other primary alternative to CAR T-cell therapy among children with r/r ALL, with the fundamental consideration being whether or not CAR T-cells represent a replacement for HSCT, or an adjunctive treatment to facilitate a negative pre-transplant MRD status (and if an adjunctive treatment, the optimal timing of CAR T-cell therapy in relation to HSCT).

The use of CAR T-cell therapy as a stand-alone replacement for HSCT is predicated upon the long-term, *in vivo* persistence of the CAR T-cells. As is discussed subsequently, CAR T-cell persistence is vital for ensuring ongoing remission/cure. This approach is attractive in that it allows avoidance of the substantial toxicities and late effects of HSCT. The converse combinatorial approach, whereby CAR T-cells are infused prior to planned HSCT, envisions them not as a curative approach in their own right, but rather as a means to facilitate a deep remission pre-transplant. Given the high cost of both therapies, this approach is economically more challenging; HSCT-specific toxicities are also not avoided. Most importantly, a subset of patients – those who would otherwise be cured by CAR T-cell therapy alone - may undergo unnecessary HSCTs, exposing them to the risks but none of the benefits associated with this treatment modality. But definitive proof of cure by CAR T-cell therapy is difficult to predict for any particular patient, though surrogate biomarkers such as loss of B-cell aplasia or emergence of next-generation sequencing minimal residual disease (NGS-MRD) are being explored as early markers of impending relapse. It is also important to note that neither monitoring approach would be helpful for CD19-negative relapses.

By carefully considering patient/disease/treatment characteristics, it may be possible, in the future, to construct an individualized decision matrix for each patient. By doing so, the relative efficacy of CAR T-cell therapy alone may be estimated, versus the risk of needing to proceed to consolidative HSCT or to undergo an HSCT following post-CAR T-infusion relapse. Unfortunately, there is a dearth of prospective and/or randomized studies directly comparing the two therapies, or the optimal strategies to integrate them. Several factors are, however, known to be predictive of CAR T-cell efficacy, and may be of use in making this decision.

First, an intriguing analysis suggested that superior outcomes may be achieved via the utilization of higher infused cell doses.^98,99^ Although not powered to specifically detect dose-response relationships, nor to suggest specific target doses, a relationship between the number of infused CAR T-cells and patient outcomes cannot be excluded based on the present studies. For reference, tisagenlecleucel’s recommended dosing is 0.2–5.0 × 10^6^/kg for patients below 50 kg, 0.1–2.5 × 10^8^ for patients weighing >50 kg. Second, CAR T-cell expansion has been correlated with response to therapy, with higher peak concentrations and larger AUC in the 28 days following infusion both being associated with superior outcomes.^98^ Third, a high tumor burden at the time of infusion appears to be correlated with lower EFS and OS, when compared to either undetectable marrow disease or <5% bone marrow blasts; in particular CD19-negative relapses are more common among those with a higher marrow burden.^52,97,100^ Fourth, high-risk cytogenetics (particularly lysine methyltransferase 2A (*KMT2A*) may be predictive of a higher rate of post-CAR T-cell relapse and, in particular, lineage-switching to AML,^101^ though this association has not been consistently observed, and some studies show no difference between patients with versus without high-risk cytogenetics.^100^ Consolidative HSCT in patients with TP53 mutations does not appear to improve outcomes.^102^

Conversely, several other factors have been found to be predictive of CAR T-cell therapy failure. First, persistence of B-cell aplasia is a useful surrogate marker for CAR T-cell persistence, as B-cell recovery prior to 6-months has been directly associated with loss of CAR T-cells.^98^ Patients with B-cell recovery prior to 6 months therefore appear to be at heightened risk of relapse due to failure of CAR T-cell persistence. Second, depth of remission, as measured by NGS-MRD, is predictive of relapse.^103^ In particular, any detectable NGS-MRD within the first 28 days appears highly correlated with a dramatic and significant reduction in 2-year EFS (68% versus 23%) and OS (84% versus. 47%).^104^ Third, loss of the CD19 target antigen on lymphoblasts is also predictive of relapse, and may be seen even in the presence of persistent B-cell aplasia and/or the presence of CAR T-cells.^100^ Many of the above findings are elucidated in further detail in a recent review article which explores the CAR T-cell versus HSCT dichotomy in greater detail.^103^

## ALTERNATIVES TO AUTOLOGOUS CAR T-CELL THERAPY: ALLOGENEIC CAR T-CELLS

The use of allogeneic CAR T-cells in children is in its relative infancy compared to autologous CAR T-cell therapy. They hold an undeniable appeal however – the availability of “off the shelf” CAR T-cells would allow near-immediate clinical use, with a vastly simplified logistics chain (e.g. no requirement for leukapheresis or transport of collected cells), and no collection-related difficulties or possibility of malignant product contamination.

The UCART19 is one such product, and has been evaluated in children/adolescents with r/r B-ALL.^105^ It is an allogeneic, CD19-directed CAR T-cell product which has been modified to not express CD52 or the TCR alpha-chain, thereby minimizing the risk of graft versus host disease (GVHD). It was first administered to two children with r/r infant B-ALL,^106^ both of whom were able to achieve remission and undergo allogeneic HSCT, and both of whom remained alive/in remission at least 4 years post-infusion.^105^ UCART19 was subsequently evaluated in a series of phase 1 studies, one of which examined pediatric patients.^105^ That study (“PALL”) included 7 heavily pre-treated children (median 4 prior lines of therapy (range 3-6), 3 with prior HSCT, 3 with prior CD19-directed therapies), with a median marrow disease burden of 6% (range 0.0-80.0). Of these 7 participants, all achieved an initial complete response. One was subsequently lost to follow up, but all the other 6 underwent allogenic HSCT, with 2 (29%) remaining alive and in remission past 2 years of follow-up, and 4 (57%) dying (3 of progressive/relapsed disease, and 1 from infectious complications following HSCT). Toxicity data were not reported separately for the pediatric participants. However, for the entire cohort (7 children, 14 adults) 91% (19 patients) experienced CRS, with 3 of these (14%) being grade 3-4. Grade 1-2 neurotoxicity was seen in 38% (8 patients), and 2 patients (10%) experienced grade 1 acute cutaneous GVHD. The safety profile was considered to be acceptable. Further development of this platform appears to be underway, but does not appear to include pediatric clinical trials (109).

Allogenic CAR T-cells have also been explored in T-cell ALL/ Lymphoblastic lymphoma (T-ALL/LBL), by targeting CD7.^107^ This product (WU-CART-007) is currently under evaluation in patients aged 12 years and up (NCT04984356). Initial phase 1 / 2 clinical trial results have recently been released,^107^ but do not include any participants younger than age 20 (median age 33.5 years (range 20-68)). Results from the initial 18 patients showed dose-dependent activity and responses, with the 12 evaluable patients having a 58% composite CR rate, and 2 being able to proceed to consolidative allogenic HSCT. The safety profile was largely in keeping with autologous CAR T-cell products: CRS was observed in 14 of 18 (78%) recipients, with 13 of those having grade 1 or 2 CRS, and a single patient having grade 3 CRS which resolved following tocilizumab and dexamethasone use. A single case of grade 1 neurotoxicity was also seen. Importantly, no anti-donor / anti-HLA antibodies were detected, nor was there evidence of anti-drug antibodies against the CAR-construct, or graft (e.g. CAR T-cell) versus host disease.

## BEYOND CD19: ALTERNATIVE TARGETS

Among children treated with CD19-directed CAR T-cell therapies, CD19-negaive relapse is the primary cause of treatment failure, and occurs in 25-42% of patients who initially respond to treatment.^32,108^ There is therefore interest in alternative targets beyond CD19, as well in co-targeting approaches, whereby CD19 is one of several targeted antigens.

CD22-directed CAR T-cells have been explored in a phase I study of 21 children and young adults with r/r B-ALL.^109^ Participants were heavily pre-treated, with all 21 patients having previously undergone HSCT (and 2 having undergone 2 HSCTs), 15 having received prior CD19-directed CAR T-cell therapy, and 2 having received blinatumomab. Ten participants had

CD19-negative or CD19-dim lymphoblasts. Participants also had a relatively high disease burden, with a median marrow blast percentage of 70.5% (range 1%-99%). Despite this, the therapy was relatively efficacious, particularly at doses of ≥ 1 × 10^6^/kg. Among the 15 patients who received this dose or greater, 11 (73%) achieved CR, including 9 of 10 patients who had previously received CD19-directed therapies. Among the 6 patients who received the lower dose, 3×10^5^/kg, only 1 achieved CR. Toxicity was consistent with prior CD19-directed therapies, with grade 1 or 2 CRS seen in 16/21 (76%) of patients, but no grade 3/4 CRS, and 6 patients (29%) experiencing grade 1 neurological side effects, but no severe neurological toxicity. The median duration of remission was 6 months, with 3 patients remaining in remission at 21, 9, and 6 months post-infusion. All relapses were associated with reductions or loss of CD22 expression.

Based on the evident ability of lymphoblasts to downregulate CD19 or CD22 expression, a combinatorial approach targeting both antigens is promising. Two such therapies have been examined in children/young adults, both in phase I studies

The first such trial (AMELIA; NCT03289455) included 15 children with r/r B-ALL, and utilized a second generation, autologous CAR T-cell product (AUTO3) expressing both anti-CD19 and anti-CD22 CARs, while also including a tumor necrosis factor receptor (TNFR) co-stimulatory domain.^110^ Of the participants, one had received both prior CD19-directed CAR T-cells and blinatumomab therapy, and 14 had not received any prior CAR T-cell products. Seven of the 15 participants had previously undergone HSCT. The median marrow disease burden was 7.5% (range 0-90%). The product was well tolerated, with 80% (12/15) developing mild (grade 1/2) CRS, and no severe CRS seen. A total of 27% (4/15) experienced grade 1 neurotoxicity, and one patient developed grade 3 encephalopathy, which was not attributed to the CAR T-cell product. At 1-month post-infusion, 86% (13/15) of patients had achieved either CR or CR with incomplete marrow recovery. The 1-year OS and EFS were 60% and 32%, respectively, and it was theorized that relapses occurred largely due to poor long-term persistence of the AUTO 3 product (median duration of detectable CAR T-cells 91 days, range 19-571).

The second such product was tested in 20 children / young adult patients, and was a novel murine stem cell virus (MSCV)-transduced CD19/CD22-4-1BB bivalent CAR T-cell product (CD19.22.BBζ) (NCT03448393).^111^ Twelve (60%) of the participants had previously undergone HSCT, 15 (75%) had received CD19-directed therapies, including 14 (70%) who had previously received blinatumomab, and 6 (30%) who had previously received CAR T-cells. One patient was MRD negative prior to receiving the CD19.22.BBζ product, 10 (50%) were M1 (<5% blasts), and 9 (45%) were M2 (5-25% blasts). Ten (50%) patients developed CRS: 7 with grade 1/2 and 3 with grade 3/4. A single patient experienced grade 3 neurotoxicity. Twelve (60%) achieved CR at all sites, while 16 (80%) achieved marrow MRD-negative status (the discrepant 4 patients had persistence of extramedullary disease). Interestingly, 71% (10/14) of the CAR-naive patients achieved CR, while only 33% (2/6) patients who had previously received CAR T-cell therapy achieved CR (although this was not a significant difference; p=0.16). Among the 12 patients who achieved CR, the 6- and 12-month RFS were 80.1% (95% CI: 42.4-94.9%) and 57.7% (95% CI: 22.1%-81.9%), respectively. Poor CAR T-cell persistence was felt to play a role in relapse in this study as well, and efforts to improve upon this are ongoing.

## Implementation of Outpatient CAR-T Therapy in Children

Familiarity and experience with CAR-T therapy in children has grown rapidly in recent years. In particular, use of the FDA-approved tisagenlecleucel for r/r ALL is increasing. Although age- and indication-specific data are not readily available, nearly 7,000 patients were reported to have received tisagenlecleucel as of mid-2022.^112^ As a result of this expanding usage, and of advances in both recognition and management of CAR-T-associated toxicities, an increasing proportion of patients are receiving CAR-T products in the outpatient setting and subsequently being managed in the community.^113^ In this context, planned admission to the inpatient setting is envisioned as ideally being needed only for those at high risk of experiencing adverse events, and not as a matter of routine.

A number of studies have examined CAR T-cell therapy in the outpatient setting.^114–122^ These have focused mostly on adults, but a small number of children have also been included (without specific pediatric sub-analyses reported). Broadly, these studies found highly variable rates of admission following outpatient infusions and/or outpatient post-infusion monitoring, ranging from 36-88% of infused patients, with a great degree of variability depending on both disease indication and CAR T-cell product used. Despite the high rates of readmission, however, there has been negligible evidence that outpatient CAR T-cell administration is associated with clinically significant delays in care or worse outcomes, while affording substantial economic and quality of life benefits. A full review and summary of these studies’ specific findings has recently been published.^113^ Studies specifically assessing outpatient CAR T-cell administration in pediatric patients are lacking, although a small number of children have been included in larger series examining outpatient infusion/management.^120–122^ Conversely, in the pivotal trial which led to tisagenlecleucel’s approval for pediatric r/r ALL, 76% of the children received their CAR-T-cell infusions as inpatients.^32^ The ideal setting (e.g., inpatient or outpatient) therefore must be individualized for a given patient, depending on that patient’s risk profile, the toxicity profile of the CAR T-cell product, and the ability of the clinical team to facilitate such care.

Guidelines for outpatient administration of CAR T-cells, including in children, have recently been published.^113^ Briefly, these recommend specific monitoring strategies and the use of validated scoring tools, as well as outline the prerequisites for safe outpatient care, including^1^:

reliable caregivers,^2^ who have been educated about both CAR T-cell toxicities and^3^ general oncologic complications. Reliable access to transportation^4^ is also required, as is^5^ a reliable 24/7 means of triage and communication,^6^ with rapid access to a higher level of care, including intensive-care level management. Finally,^7^ regular outpatient monitoring should occur, ideally multiple times per week, throughout the highest risk period following infusion.

## FUTURE DIRECTIONS

This review has necessarily focused on the use of CAR T-cell therapy in pediatric r/r B-ALL. As noted, the only FDA-approved product in this context is tisagenlecleucel, although trials of other CD19-directed therapies are ongoing, both in r/r B-ALL and in other B-cell-derived malignancies. This relates largely to the unique features of CD19 which make it an attractive target. However, a number of promising CAR T-cell products which target widely disparate antigens are currently in development and/or under evaluation in pediatric clinical trials. There is particular interest in CAR T-cell therapies to address neuroblastoma, tumors of the CNS, and AML, given the still-disappointing outcomes for children with these conditions, particularly in the context of relapsed or refractory disease. Although it is not our intention to provide a comprehensive overview of these trials, a number of recent advances are worth discussing.

## ACUTE MYELOID LEUKEMIA

In pediatric AML, a key challenge is the lack of an antigenic target specific to myeloblasts. The most promising targets, CD33, CD123, CD135 (e.g. FLT3; FMS-like Tyrosine Kinase 3), and CLL-1 (C-type lectin-like molecule-1), are expressed in approximately 90-99%, 50-78%, 54-92%, and 78-92% of AML cases, respectively.^123^ Many of these antigens are also expressed on hematopoietic stem cells and/or early multi-lineage hematopoietic progenitors (although, generally, at relatively low expression levels than are seen in myeloblasts).^124,125^ There are, therefore, greater potential on-target toxicities involved in targeting these antigens than are seen in targeting CD19. Several review articles have recently been published^123,126^ which summarize the recent pre-clinical and clinical data surrounding the use of CAR T-cell therapy in AML and the targeting of these antigens. Thus far, the results of clinical trials have been relatively disappointing, particularly when compared to those seen in B-ALL. One of the aforementioned recent reviews^126^ summarizes all current and pending clinical trials of CAR T-cells in AML, including a number in pediatric patients,^127,128^ which are highlighted below.

The first of these^128^ utilized a CLL1-directed CAR T-cell construct with a 4-1BB costimulatory domain, which was administered to 8 children with r/r AML. All participants experienced grade 1-2 CRS, but other toxicities were minimal. Six patients achieved MRD-negative and/or CR states (2 experienced progressive disease), and were able to subsequently undergo HSCT, with 4 of these 6 remaining in CR/MRD-negative status at the time the data were presented. A separate study^127^ included 7 children with r/r AML and administered one of two different CLL1-directed CAR T-cell therapies, either a 4-1BB-based costimulatory approach (3 patients), or a CD28/CD27-based costimulatory construct (4 patients). All children experienced grade 1 or 2 CRS, and 1 patient (in the 4-1BB arm) experienced grade 2 neurotoxicity. Three of 4 children in the CD28/27 arm achieved CR, as did 2 of 3 in the 4-1BB arm; the 1-year OS was 57% (e.g. 4/7 patients). Three patients subsequently underwent HSCT, and, at the time of publication, 1 patient remained alive. Additionally, at least 2 additional trials are currently planned or actively enrolling pediatric patients.^126^

## HIGH-RISK NEUROBLASTOMA

High-risk neuroblastoma also appears to be amenable to treatment with CAR T-cells, particularly those directed against GD2. The largest and most promising investigation was recently published.^129^ Specifically, 27 children were enrolled in a phase I/II study, with 12 having refractory disease, 14 relapsed disease, and 1who had a complete response following completion first-line therapy. Patients were administered third-generation GD2-directed CAR T-cells which also featured inducible caspase 9 “suicide gene” (GD2-CART01). All enrolled patients were able to receive CAR T-cells. GD2-CART01 appeared to demonstrate tolerable toxicity, with CRS being seen in 74% (20 / 27 patients), mostly as grade 1 or 2 CRS, and a single patient experiencing grade 3 CRS, which resolved upon receiving tocilizumab. No neurotoxicity events were seen, and 22% (6 / 27) experienced grade 1 or 2 pain, likely secondary to GD2-targetting. The only common grade 3 or 4 toxicities were hematological in nature (anemia/thrombocytopenia/neutropenia), although 26% (7 / 27) also experienced hepatoxicity (with 5 of these 7 events being a worsening of existing hepatic dysfunction rather than a new finding). Median GD2-CART01 persistence was 3 months, and 75% of the evaluable patients had persistence for 3 months or longer. Response rates were also promising, with a 33% (8 / 27) CR rate (and maintained CR in 1 patient already in CR). At a median 1.7 year follow up, 19% of recipients (5 / 27) remained in CR. Eight (30%) experienced a partial response (PR), and 10 (37%) had either stable disease (SD) (by International Neuroblastoma Response Criteria; INRC) or showed no response (by Immune-Related Response Criteria; IRRC), giving a 63% (17/27) overall objective response rate. At 3-years post-infusion, the OS was 40% and the EFS was 27%. Among the children receiving the recommended dose of 10 x10^6^/kg, 3-year OS and EFS 60% and 36%, respectively. The authors reported that further investigations are now underway and seek to integrate the GD2-CART01 CAR T-therapy into the treatment of high-risk neuroblastoma.

## CENTRAL NERVOUS SYSTEM TUMORS

Tumors of the CNS are perhaps the most challenging targets for CAR T-cell therapy, owing to several factors.^130,131^ First, the difficulty inherent to accessing the CNS imposed by the presence of the blood brain barrier (BBB). Second, the heightened risk of adverse events related specifically to CAR T-cell therapies, as well as the heightened risk of those specific toxicities occurring in their more severe forms, imposed by the location of the primary disease within the CNS. For instance, while neurotoxicity may be seen in systemically administered CD19-directed therapies, the risk of severe neurotoxicity, as well as the potential consequences of that neurotoxicity, may be increased when the target antigen exists primarily within the CNS. Third, the intensely immunosuppressive tumor microenvironment found within many primary CNS tumors^132^ may impair CAR T-cell efficacy.^133^ Finally, intra-and-intertumoral heterogeneity impairs target selection. Even within an individual tumor, or between patients with the same type of tumor, the expression levels or even presence/absence of specific antigens may vary.^134^ These challenges, as well as some of the means by which they may be overcome have recently been discussed in a pair of excellent review articles.^130,131^ Despite these limitations, some early evidence of success in pediatric clinical trials has been seen.

One notable recent example of progress in the use of CAR T-cells to treat pediatric CNS tumors has been the use of intracranially-infused (e.g. locoregional) B7-H3-directed CAR T-cells for children with r/r CNS tumors or diffuse intrinsic pontine gliomas (DIPGs), e.g. the BrainChild-03 clinical trial (NCT04185038).^135^ Data are available from the first three children enrolled on this trial, all three of whom were diagnosed with DIPGs. All three received weekly intraventricular infusions of 1 × 10^7^ B7-H3 CAR T-cells, for a total of 10, 12, and 18 infusions. As of publication, two patients experienced disease progression but were alive at 12- and 17-months post-infusion, and 26- and 36-months following initial diagnosis, while the third patient experienced stable disease and remains alive at 12 months post-infusion and 26 months post-diagnosis. All children experienced headaches, nausea, vomiting, and fevers beginning approximately 24 hours following infusions, but notably, all returned to baseline within 72 hours, and no patient experienced any dose-limiting toxicities, nor did any required pharmacologic intervention. Separately, BrainChild-01 (NCT03500991) utilizes a CAR T-cell targeting HER2 in children/young adult patients with r/r CNS tumors which are HER2-positive,^136^ while BrainChild-02 ((NCT03638167) utilizes an EGFR806-directed CAR T-cell among adolescent/young-adult patients with r/r CNS tumors expressing a tumor-specific untethered EGFR epitope.^137^ All three BrainChild clinical trials are ongoing, although only BrainChild-03 is actively recruiting patients.

## CONCLUSIONS

CD19-directed CAR T-cell products have rapidly moved from being an experimental, unproven therapy into a relatively widely available, well-characterized, and potentially curative option for pediatric patients with r/r B-cell ALL. CAR T-cell products directed against other antigens are continually being developed and evaluated in numerous clinical trials. Although there are notable obstacles which must be overcome, there is much reason to be optimistic that these therapies will eventually bring similar revolutionary improvements for patients with a diverse array of malignant conditions. While robust data exist about risks of long-term toxicity from traditional chemotherapy, less is known about those risks following a genetically modified cellular therapy. As CAR T-cell therapy continues to mature as a treatment modality, it will be essential that these patients continue to be followed for long-term late effects and outcomes well into the future.

## References

[ref-288343] Bhojwani Deepa, Yang Jun J., Pui Ching-Hon (2015). Biology of Childhood Acute Lymphoblastic Leukemia. Pediatr Clin North Am.

[ref-288344] Siegel Rebecca L., Miller Kimberly D., Fuchs Hannah E., Jemal Ahmedin (2021). Cancer Statistics, 2021. CA: A Cancer Journal for Clinicians.

[ref-288345] Liu Yuan-Fang, Wang Bai-Yan, Zhang Wei-Na, Huang Jin-Yan, Li Ben-Shang, Zhang Ming, Jiang Lu, Li Jian-Feng, Wang Ming-Jie, Dai Yu-Jun, Zhang Zi-Guan, Wang Qiang, Kong Jie, Chen Bing, Zhu Yong-Mei, Weng Xiang-Qin, Shen Zhi-Xiang, Li Jun-Min, Wang Jin, Yan Xiao-Jing, Li Yan, Liang Ying-Min, Liu Li, Chen Xie-Qun, Zhang Wang-Gang, Yan Jin-Song, Hu Jian-Da, Shen Shu-Hong, Chen Jing, Gu Long-Jun, Pei Deqing, Li Yongjin, Wu Gang, Zhou Xin, Ren Rui-Bao, Cheng Cheng, Yang Jun J., Wang Kan-Kan, Wang Sheng-Yue, Zhang Jinghui, Mi Jian-Qing, Pui Ching-Hon, Tang Jing-Yan, Chen Zhu, Chen Sai-Juan (2016). Genomic Profiling of Adult and Pediatric B-cell Acute Lymphoblastic Leukemia. EBioMedicine.

[ref-288346] Kochenderfer James N., Dudley Mark E., Feldman Steven A., Wilson Wyndham H., Spaner David E., Maric Irina, Stetler-Stevenson Maryalice, Phan Giao Q., Hughes Marybeth S., Sherry Richard M., Yang James C., Kammula Udai S., Devillier Laura, Carpenter Robert, Nathan Debbie-Ann N., Morgan Richard A., Laurencot Carolyn, Rosenberg Steven A. (2012). B-cell depletion and remissions of malignancy along with cytokine-associated toxicity in a clinical trial of anti-CD19 chimeric-antigen-receptor–transduced T cells. Blood.

[ref-288347] Brentjens Renier J., Rivière Isabelle, Park Jae H., Davila Marco L., Wang Xiuyan, Stefanski Jolanta, Taylor Clare, Yeh Raymond, Bartido Shirley, Borquez-Ojeda Oriana, Olszewska Malgorzata, Bernal Yvette, Pegram Hollie, Przybylowski Mark, Hollyman Daniel, Usachenko Yelena, Pirraglia Domenick, Hosey James, Santos Elmer, Halton Elizabeth, Maslak Peter, Scheinberg David, Jurcic Joseph, Heaney Mark, Heller Glenn, Frattini Mark, Sadelain Michel (2011). Safety and persistence of adoptively transferred autologous CD19-targeted T cells in patients with relapsed or chemotherapy refractory B-cell leukemias. Blood.

[ref-288348] Lemal Richard, Tournilhac Olivier (2019). State-of-the-art for CAR T-cell therapy for chronic lymphocytic leukemia in 2019. Journal for Immunotherapy of Cancer.

[ref-288349] Todorovic Zeljko, Todorovic Dusan, Markovic Vladimir, Ladjevac Nevena, Zdravkovic Natasa, Djurdjevic Predrag, Arsenijevic Nebojsa, Milovanovic Marija, Arsenijevic Aleksandar, Milovanovic Jelena (2022). CAR T Cell Therapy for Chronic Lymphocytic Leukemia: Successes and Shortcomings. Current Oncology.

[ref-288350] Schuster Stephen J., Bishop Michael R., Tam Constantine S., Waller Edmund K., Borchmann Peter, McGuirk Joseph P., Jäger Ulrich, Jaglowski Samantha, Andreadis Charalambos, Westin Jason R., Fleury Isabelle, Bachanova Veronika, Foley S. Ronan, Ho P. Joy, Mielke Stephan, Magenau John M., Holte Harald, Pantano Serafino, Pacaud Lida B., Awasthi Rakesh, Chu Jufen, Anak Özlem, Salles Gilles, Maziarz Richard T. (2019). Tisagenlecleucel in Adult Relapsed or Refractory Diffuse Large B-Cell Lymphoma. New England Journal of Medicine.

[ref-288351] Kanate Abraham S., Majhail Navneet, DeFilipp Zachariah, Dhakal Binod, Dholaria Bhagirathbhai, Hamilton Betty, Herrera Alex F., Inamoto Yoshihiro, Jain Tania, Perales Miguel-Angel, Carpenter Paul A., Hamadani Mehdi (2023). Updated Indications for Immune Effector Cell Therapy: 2023 Guidelines from the American Society for Transplantation and Cellular Therapy. Transplantation and Cellular Therapy.

[ref-288352] Home - ClinicalTrials.gov.

[ref-288353] Hines Melissa R., Knight Tristan E., McNerney Kevin O., Leick Mark B., Jain Tania, Ahmed Sairah, Frigault Matthew J., Hill Joshua A., Jain Michael D., Johnson William T., Lin Yi, Mahadeo Kris M., Maron Gabriela M., Marsh Rebecca A., Neelapu Sattva S., Nikiforow Sarah, Ombrello Amanda K., Shah Nirav N., Talleur Aimee C., Turicek David, Vatsayan Anant, Wong Sandy W., Maus Marcela V., Komanduri Krishna V., Berliner Nancy, Henter Jan-Inge, Perales Miguel-Angel, Frey Noelle V., Teachey David T., Frank Matthew J., Shah Nirali N. (2023). Immune Effector Cell-Associated Hemophagocytic Lymphohistiocytosis-Like Syndrome. Transplantation and Cellular Therapy.

[ref-288354] Kershaw Michael H., Westwood Jennifer A., Parker Linda L., Wang Gang, Eshhar Zelig, Mavroukakis Sharon A., White Donald E., Wunderlich John R., Canevari Silvana, Rogers-Freezer Linda, Chen Clara C., Yang James C., Rosenberg Steven A., Hwu Patrick (2006). A Phase I Study on Adoptive Immunotherapy Using Gene-Modified T Cells for Ovarian Cancer. Clinical Cancer Research.

[ref-288355] Lamers Cor H.J., Sleijfer Stefan, Vulto Arnold G., Kruit Wim H.J., Kliffen Mike, Debets Reno, Gratama Jan W., Stoter Gerrit, Oosterwijk Egbert (2006). Treatment of Metastatic Renal Cell Carcinoma With Autologous T-Lymphocytes Genetically Retargeted Against Carbonic Anhydrase IX: First Clinical Experience. Journal of Clinical Oncology.

[ref-288356] Till Brian G., Jensen Michael C., Wang Jinjuan, Chen Eric Y., Wood Brent L., Greisman Harvey A., Qian Xiaojun, James Scott E., Raubitschek Andrew, Forman Stephen J., Gopal Ajay K., Pagel John M., Lindgren Catherine G., Greenberg Philip D., Riddell Stanley R., Press Oliver W. (2008). Adoptive immunotherapy for indolent non-Hodgkin lymphoma and mantle cell lymphoma using genetically modified autologous CD20-specific T cells. Blood.

[ref-288357] Jensen Michael C., Popplewell Leslie, Cooper Laurence J., DiGiusto David, Kalos Michael, Ostberg Julie R., Forman Stephen J. (2010). Antitransgene Rejection Responses Contribute to Attenuated Persistence of Adoptively Transferred CD20/CD19-Specific Chimeric Antigen Receptor Redirected T Cells in Humans. Biology of Blood and Marrow Transplantation.

[ref-288358] Porter David L., Levine Bruce L., Kalos Michael, Bagg Adam, June Carl H. (2011). Chimeric Antigen Receptor–Modified T Cells in Chronic Lymphoid Leukemia. New England Journal of Medicine.

[ref-288359] Pettitt David, Arshad Zeeshaan, Smith James, Stanic Tijana, Holländer Georg, Brindley David (2018). CAR-T Cells: A Systematic Review and Mixed Methods Analysis of the Clinical Trial Landscape. Molecular Therapy.

[ref-288360] Singh Nathan, Frey Noelle V., Grupp Stephan A., Maude Shannon L (2016). CAR T Cell Therapy in Acute Lymphoblastic Leukemia and Potential for Chronic Lymphocytic Leukemia. Current Treatment Options in Oncology.

[ref-288361] Gill Saar, Maus Marcela V., Porter David L. (2016). Chimeric antigen receptor T cell therapy: 25years in the making. Blood Reviews.

[ref-288362] Braendstrup Peter, Levine Bruce L., Ruella Marco (2020). The long road to the first FDA-approved gene therapy: chimeric antigen receptor T cells targeting CD19. Cytotherapy.

[ref-288363] Savoldo Barbara, Ramos Carlos Almeida, Liu Enli, Mims Martha P., Keating Michael J., Carrum George, Kamble Rammurti T., Bollard Catherine M., Gee Adrian P., Mei Zhuyong, Liu Hao, Grilley Bambi, Rooney Cliona M., Heslop Helen E., Brenner Malcolm K., Dotti Gianpietro (2011). CD28 costimulation improves expansion and persistence of chimeric antigen receptor–modified T cells in lymphoma patients. Journal of Clinical Investigation.

[ref-288364] Long Adrienne H, Haso Waleed M, Shern Jack F, Wanhainen Kelsey M, Murgai Meera, Ingaramo Maria, Smith Jillian P, Walker Alec J, Kohler M Eric, Venkateshwara Vikas R, Kaplan Rosandra N, Patterson George H, Fry Terry J, Orentas Rimas J, Mackall Crystal L (2015). 4-1BB costimulation ameliorates T cell exhaustion induced by tonic signaling of chimeric antigen receptors. Nature Medicine.

[ref-288365] Milone Michael C., Fish Jonathan D., Carpenito Carmine, Carroll Richard G., Binder Gwendolyn K., Teachey David, Samanta Minu, Lakhal Mehdi, Gloss Brian, Danet-Desnoyers Gwenn, Campana Dario, Riley James L., Grupp Stephan A., June Carl H. (2009). Chimeric Receptors Containing CD137 Signal Transduction Domains Mediate Enhanced Survival of T Cells and Increased Antileukemic Efficacy In Vivo. Molecular Therapy.

[ref-288366] Carpenito Carmine, Milone Michael C., Hassan Raffit, Simonet Jacqueline C., Lakhal Mehdi, Suhoski Megan M., Varela-Rohena Angel, Haines Kathleen M., Heitjan Daniel F., Albelda Steven M., Carroll Richard G., Riley James L., Pastan Ira, June Carl H. (2009). Control of large, established tumor xenografts with genetically retargeted human T cells containing CD28 and CD137 domains. Proceedings of the National Academy of Sciences.

[ref-288367] Zhong Xiao-Song, Matsushita Maiko, Plotkin Jason, Riviere Isabelle, Sadelain Michel (2010). Chimeric Antigen Receptors Combining 4-1BB and CD28 Signaling Domains Augment PI3kinase/AKT/Bcl-XL Activation and CD8+ T Cell–mediated Tumor Eradication. Molecular Therapy.

[ref-288368] Tammana Syam, Huang Xin, Wong Marianna, Milone Michael C., Ma Linan, Levine Bruce L., June Carl H., Wagner John E., Blazar Bruce R., Zhou Xianzheng (2010). 4-1BB and CD28 signaling plays a synergistic role in redirecting umbilical cord blood T cells against B-cell malignancies. Human Gene Therapy.

[ref-288369] Tokarew Nicholas, Ogonek Justyna, Endres Stefan, von Bergwelt-Baildon Michael, Kobold Sebastian (2018). Teaching an old dog new tricks: next-generation CAR T cells. British Journal of Cancer.

[ref-288370] Kagoya Yuki, Tanaka Shinya, Guo Tingxi, Anczurowski Mark, Wang Chung-Hsi, Saso Kayoko, Butler Marcus O, Minden Mark D, Hirano Naoto (2018). A novel chimeric antigen receptor containing a JAK–STAT signaling domain mediates superior antitumor effects. Nature Medicine.

[ref-288371] Grupp Stephan A., Kalos Michael, Barrett David, Aplenc Richard, Porter David L., Rheingold Susan R., Teachey David T., Chew Anne, Hauck Bernd, Wright J. Fraser, Milone Michael C., Levine Bruce L., June Carl H. (2013). Chimeric Antigen Receptor–Modified T Cells for Acute Lymphoid Leukemia. New England Journal of Medicine.

[ref-288372] Lee Daniel W., Santomasso Bianca D., Locke Frederick L., Ghobadi Armin, Turtle Cameron J., Brudno Jennifer N., Maus Marcela V., Park Jae H., Mead Elena, Pavletic Steven, Go William Y., Eldjerou Lamis, Gardner Rebecca A., Frey Noelle, Curran Kevin J., Peggs Karl, Pasquini Marcelo, DiPersio John F., van den Brink Marcel R.M., Komanduri Krishna V., Grupp Stephan A., Neelapu Sattva S. (2019). ASTCT Consensus Grading for Cytokine Release Syndrome and Neurologic Toxicity Associated with Immune Effector Cells. Biology of Blood and Marrow Transplantation.

[ref-288373] Maude Shannon L., Frey Noelle, Shaw Pamela A., Aplenc Richard, Barrett David M., Bunin Nancy J., Chew Anne, Gonzalez Vanessa E., Zheng Zhaohui, Lacey Simon F., Mahnke Yolanda D., Melenhorst Jan J., Rheingold Susan R., Shen Angela, Teachey David T., Levine Bruce L., June Carl H., Porter David L., Grupp Stephan A. (2014). Chimeric Antigen Receptor T Cells for Sustained Remissions in Leukemia. New England Journal of Medicine.

[ref-288374] Maude Shannon L., Laetsch Theodore W., Buechner Jochen, Rives Susana, Boyer Michael, Bittencourt Henrique, Bader Peter, Verneris Michael R., Stefanski Heather E., Myers Gary D., Qayed Muna, De Moerloose Barbara, Hiramatsu Hidefumi, Schlis Krysta, Davis Kara L., Martin Paul L., Nemecek Eneida R., Yanik Gregory A., Peters Christina, Baruchel Andre, Boissel Nicolas, Mechinaud Francoise, Balduzzi Adriana, Krueger Joerg, June Carl H., Levine Bruce L., Wood Patricia, Taran Tetiana, Leung Mimi, Mueller Karen T., Zhang Yiyun, Sen Kapildeb, Lebwohl David, Pulsipher Michael A., Grupp Stephan A. (2018). Tisagenlecleucel in Children and Young Adults with B-Cell Lymphoblastic Leukemia. New England Journal of Medicine.

[ref-288375] Laetsch Theodore W., Maude Shannon L., Rives Susana, Hiramatsu Hidefumi, Bittencourt Henrique, Bader Peter, Baruchel André, Boyer Michael, De Moerloose Barbara, Qayed Muna, Buechner Jochen, Pulsipher Michael A., Myers Gary Douglas, Stefanski Heather E., Martin Paul L., Nemecek Eneida, Peters Christina, Yanik Gregory, Khaw Seong Lin, Davis Kara L., Krueger Joerg, Balduzzi Adriana, Boissel Nicolas, Tiwari Ranjan, O'Donovan Darragh, Grupp Stephan A. (2023). Three-Year Update of Tisagenlecleucel in Pediatric and Young Adult Patients With Relapsed/Refractory Acute Lymphoblastic Leukemia in the ELIANA Trial. Journal of Clinical Oncology.

[ref-288376] O'Leary Maura C., Lu Xiaobin, Huang Ying, Lin Xue, Mahmood Iftekhar, Przepiorka Donna, Gavin Denise, Lee Shiowjen, Liu Ke, George Bindu, Bryan Wilson, Theoret Marc R., Pazdur Richard (2019). FDA Approval Summary: Tisagenlecleucel for Treatment of Patients with Relapsed or Refractory B-cell Precursor Acute Lymphoblastic Leukemia. Clinical Cancer Research.

[ref-288377] Munshi Nikhil C., Anderson Larry D., Jr., Shah Nina, Madduri Deepu, Berdeja Jesús, Lonial Sagar, Raje Noopur, Lin Yi, Siegel David, Oriol Albert, Moreau Philippe, Yakoub-Agha Ibrahim, Delforge Michel, Cavo Michele, Einsele Hermann, Goldschmidt Hartmut, Weisel Katja, Rambaldi Alessandro, Reece Donna, Petrocca Fabio, Massaro Monica, Connarn Jamie N., Kaiser Shari, Patel Payal, Huang Liping, Campbell Timothy B., Hege Kristen, San-Miguel Jesús (2021). Idecabtagene Vicleucel in Relapsed and Refractory Multiple Myeloma. New England Journal of Medicine.

[ref-288378] Berdeja Jesus G, Madduri Deepu, Usmani Saad Z, Jakubowiak Andrzej, Agha Mounzer, Cohen Adam D, Stewart A Keith, Hari Parameswaran, Htut Myo, Lesokhin Alexander, Deol Abhinav, Munshi Nikhil C, O'Donnell Elizabeth, Avigan David, Singh Indrajeet, Zudaire Enrique, Yeh Tzu-Min, Allred Alicia J, Olyslager Yunsi, Banerjee Arnob, Jackson Carolyn C, Goldberg Jenna D, Schecter Jordan M, Deraedt William, Zhuang Sen Hong, Infante Jeffrey, Geng Dong, Wu Xiaoling, Carrasco-Alfonso Marlene J, Akram Muhammad, Hossain Farah, Rizvi Syed, Fan Frank, Lin Yi, Martin Thomas, Jagannath Sundar (2021). Ciltacabtagene autoleucel, a B-cell maturation antigen-directed chimeric antigen receptor T-cell therapy in patients with relapsed or refractory multiple myeloma (CARTITUDE-1): a phase 1b/2 open-label study. The Lancet.

[ref-288379] Sun Baolan, Wu Youjia, Wu Lihui, Cui Ming, Ni Hongbing, Yang Zhiping, Xu Meiyu, Wang Huimin (2014). Raised expression of APRIL in Chinese children with acute lymphoblastic leukemia and its clinical implications. J Pediatr HematolOncol.

[ref-288380] Dogan Ahmet, Siegel David, Tran Nguyet, Fu Alan, Fowler Jessica, Belani Rajesh, Landgren Ola (2020). B-cell maturation antigen expression across hematologic cancers: a systematic literature review. Blood Cancer Journal.

[ref-288381] Neelapu Sattva S., Locke Frederick L., Bartlett Nancy L., Lekakis Lazaros J., Miklos David B., Jacobson Caron A., Braunschweig Ira, Oluwole Olalekan O., Siddiqi Tanya, Lin Yi, Timmerman John M., Stiff Patrick J., Friedberg Jonathan W., Flinn Ian W., Goy Andre, Hill Brian T., Smith Mitchell R., Deol Abhinav, Farooq Umar, McSweeney Peter, Munoz Javier, Avivi Irit, Castro Januario E., Westin Jason R., Chavez Julio C., Ghobadi Armin, Komanduri Krishna V., Levy Ronald, Jacobsen Eric D., Witzig Thomas E., Reagan Patrick, Bot Adrian, Rossi John, Navale Lynn, Jiang Yizhou, Aycock Jeff, Elias Meg, Chang David, Wiezorek Jeff, Go William Y. (2017). Axicabtagene Ciloleucel CAR T-Cell Therapy in Refractory Large B-Cell Lymphoma. New England Journal of Medicine.

[ref-288382] Locke Frederick L, Ghobadi Armin, Jacobson Caron A, Miklos David B, Lekakis Lazaros J, Oluwole Olalekan O, Lin Yi, Braunschweig Ira, Hill Brian T, Timmerman John M, Deol Abhinav, Reagan Patrick M, Stiff Patrick, Flinn Ian W, Farooq Umar, Goy Andre, McSweeney Peter A, Munoz Javier, Siddiqi Tanya, Chavez Julio C, Herrera Alex F, Bartlett Nancy L, Wiezorek Jeffrey S, Navale Lynn, Xue Allen, Jiang Yizhou, Bot Adrian, Rossi John M, Kim Jenny J, Go William Y, Neelapu Sattva S (2019). Long-term safety and activity of axicabtagene ciloleucel in refractory large B-cell lymphoma (ZUMA-1): a single-arm, multicentre, phase 1–2 trial. The Lancet Oncology.

[ref-288383] Locke Frederick L., Miklos David B., Jacobson Caron A., Perales Miguel-Angel, Kersten Marie-José, Oluwole Olalekan O., Ghobadi Armin, Rapoport Aaron P., McGuirk Joseph, Pagel John M., Muñoz Javier, Farooq Umar, van Meerten Tom, Reagan Patrick M., Sureda Anna, Flinn Ian W., Vandenberghe Peter, Song Kevin W., Dickinson Michael, Minnema Monique C., Riedell Peter A., Leslie Lori A., Chaganti Sridhar, Yang Yin, Filosto Simone, Shah Jina, Schupp Marco, To Christina, Cheng Paul, Gordon Leo I., Westin Jason R. (2022). Axicabtagene Ciloleucel as Second-Line Therapy for Large B-Cell Lymphoma. New England Journal of Medicine.

[ref-288384] Masonic Cancer Center, University of Minnesota (2023). Chimeric Antigen Receptor (CAR)-T Cell Therapy for Patients With Hematologic Malignancies.

[ref-288385] Shah Bijal D, Ghobadi Armin, Oluwole Olalekan O, Logan Aaron C, Boissel Nicolas, Cassaday Ryan D, Leguay Thibaut, Bishop Michael R, Topp Max S, Tzachanis Dimitrios, O'Dwyer Kristen M, Arellano Martha L, Lin Yi, Baer Maria R, Schiller Gary J, Park Jae H, Subklewe Marion, Abedi Mehrdad, Minnema Monique C, Wierda William G, DeAngelo Daniel J, Stiff Patrick, Jeyakumar Deepa, Feng Chaoling, Dong Jinghui, Shen Tong, Milletti Francesca, Rossi John M, Vezan Remus, Masouleh Behzad Kharabi, Houot Roch (2021). KTE-X19 for relapsed or refractory adult B-cell acute lymphoblastic leukaemia: phase 2 results of the single-arm, open-label, multicentre ZUMA-3 study. The Lancet.

[ref-288386] Wang Michael, Munoz Javier, Goy Andre, Locke Frederick L., Jacobson Caron A., Hill Brian T., Timmerman John M., Holmes Houston, Jaglowski Samantha, Flinn Ian W., McSweeney Peter A., Miklos David B., Pagel John M., Kersten Marie-Jose, Milpied Noel, Fung Henry, Topp Max S., Houot Roch, Beitinjaneh Amer, Peng Weimin, Zheng Lianqing, Rossi John M., Jain Rajul K., Rao Arati V., Reagan Patrick M. (2020). KTE-X19 CAR T-Cell Therapy in Relapsed or Refractory Mantle-Cell Lymphoma. New England Journal of Medicine.

[ref-288387] Kite, A Gilead Company (2023). A Phase 1/2 Multi-Center Study Evaluating the Safety and Efficacy of KTE-X19 in Pediatric and Adolescent Subjects With Relapsed/Refractory B-precursor Acute Lymphoblastic Leukemia or Relapsed/Refractory B-Cell Non-Hodgkin Lymphoma (ZUMA-4).

[ref-288388] Wayne Alan S., Huynh Van, Hijiya Nobuko, Rouce Rayne H., Brown Patrick A., Krueger Joerg, Kitko Carrie L., Ziga Edward Dela, Hermiston Michelle L., Richards Michael K., Baruchel Andre, Schuberth Petra C., Rossi John, Zhou Lang, Goyal Lovely, Jain Rajul, Vezan Remus, Masouleh Behzad Kharabi, Lee Daniel W. (2022). Three-year results from phase I of ZUMA-4: KTE-X19 in pediatric relapsed/refractory acute lymphoblastic leukemia. Haematologica.

[ref-288389] Abramson Jeremy S, Palomba M Lia, Gordon Leo I, Lunning Matthew A, Wang Michael, Arnason Jon, Mehta Amitkumar, Purev Enkhtsetseg, Maloney David G, Andreadis Charalambos, Sehgal Alison, Solomon Scott R, Ghosh Nilanjan, Albertson Tina M, Garcia Jacob, Kostic Ana, Mallaney Mary, Ogasawara Ken, Newhall Kathryn, Kim Yeonhee, Li Daniel, Siddiqi Tanya (2020). Lisocabtagene maraleucel for patients with relapsed or refractory large B-cell lymphomas (TRANSCEND NHL 001): a multicentre seamless design study. The Lancet.

[ref-288390] Kamdar Manali, Solomon Scott R, Arnason Jon, Johnston Patrick B, Glass Bertram, Bachanova Veronika, Ibrahimi Sami, Mielke Stephan, Mutsaers Pim, Hernandez-Ilizaliturri Francisco, Izutsu Koji, Morschhauser Franck, Lunning Matthew, Maloney David G, Crotta Alessandro, Montheard Sandrine, Previtali Alessandro, Stepan Lara, Ogasawara Ken, Mack Timothy, Abramson Jeremy S (2022). Lisocabtagene maraleucel versus standard of care with salvage chemotherapy followed by autologous stem cell transplantation as second-line treatment in patients with relapsed or refractory large B-cell lymphoma (TRANSFORM): results from an interim analysis of an open-label, randomised, phase 3 trial. The Lancet.

[ref-288391] Sehgal Alison, Hoda Daanish, Riedell Peter A, Ghosh Nilanjan, Hamadani Mehdi, Hildebrandt Gerhard C, Godwin John E, Reagan Patrick M, Wagner-Johnston Nina, Essell James, Nath Rajneesh, Solomon Scott R, Champion Rebecca, Licitra Edward, Fanning Suzanne, Gupta Neel, Dubowy Ronald, D'Andrea Aleco, Wang Lei, Ogasawara Ken, Thorpe Jerill, Gordon Leo I (2022). Lisocabtagene maraleucel as second-line therapy in adults with relapsed or refractory large B-cell lymphoma who were not intended for haematopoietic stem cell transplantation (PILOT): an open-label, phase 2 study. The Lancet Oncology.

[ref-288392] Celgene (2023). A Phase 1/2, Open-label, Single Arm, Multicohort, Multicenter Trial to Evaluate the Safety and Efficacy of JCAR017 in Pediatric Subjects With Relapsed/Refractory (r/r) B-cell Acute Lymphoblastic Leukemia (B-ALL) and B-cell Non-Hodgkin Lymphoma (B-NHL)..

[ref-288393] Pasquini Marcelo C., Hu Zhen-Huan, Curran Kevin, Laetsch Theodore, Locke Frederick, Rouce Rayne, Pulsipher Michael A., Phillips Christine L., Keating Amy, Frigault Matthew J., Salzberg Dana, Jaglowski Samantha, Sasine Joshua P., Rosenthal Joseph, Ghosh Monalisa, Landsburg Daniel, Margossian Steven, Martin Paul L., Kamdar Manali K., Hematti Peiman, Nikiforow Sarah, Turtle Cameron, Perales Miguel-Angel, Steinert Patricia, Horowitz Mary M., Moskop Amy, Pacaud Lida, Yi Lan, Chawla Raghav, Bleickardt Eric, Grupp Stephan (2020). Real-world evidence of tisagenlecleucel for pediatric acute lymphoblastic leukemia and non-Hodgkin lymphoma. Blood Adv.

[ref-288394] Schultz Liora M., Baggott Christina, Prabhu Snehit, Pacenta Holly L., Phillips Christine L., Rossoff Jenna, Stefanski Heather E., Talano Julie-An, Moskop Amy, Margossian Steven P., Verneris Michael R., Myers Gary Douglas, Karras Nicole A., Brown Patrick A., Qayed Muna, Hermiston Michelle, Satwani Prakash, Krupski Christa, Keating Amy K., Wilcox Rachel, Rabik Cara A., Fabrizio Vanessa A., Rouce Rayne H., Chinnabhandar Vasant, Kunicki Michael, Barsan Valentin V., Goksenin A. Yasemin, Li Yimei, Mavroukakis Sharon, Egeler Emily, Curran Kevin J., Mackall Crystal L., Laetsch Theodore W. (2022). Disease Burden Affects Outcomes in Pediatric and Young Adult B-Cell Lymphoblastic Leukemia After Commercial Tisagenlecleucel: A Pediatric Real-World Chimeric Antigen Receptor Consortium Report. Journal of Clinical Oncology.

[ref-288395] Bader Peter, Rossig Claudia, Hutter Martin, Ayuk Francis Ayuketang, Baldus Claudia D., Bücklein Veit L., Bonig Halvard, Cario Gunnar, Einsele Hermann, Holtick Udo, Koenecke Christian, Bakhtiar Shahrzad, Künkele Annette, Meisel Roland, Müller Fabian, Müller Ingo, Penack Olaf, Rettinger Eva, Sauer Martin G., Schlegel Paul-Gerhardt, Soerensen Jan, von Stackelberg Arend, Strahm Brigitte, Hauer Julia, Feuchtinger Tobias, Jarisch Andrea (2023). CD19 CAR T cells are an effective therapy for posttransplant relapse in patients with B-lineage ALL: real-world data from Germany. Blood Adv.

[ref-288396] Barsan Valentin, Li Yimei, Prabhu Snehit, Baggott Christina, Nguyen Khanh, Pacenta Holly, Phillips Christine L., Rossoff Jenna, Stefanski Heather, Talano Julie-An, Moskop Amy, Baumeister Susanne, Verneris Michael R., Myers Gary Douglas, Karras Nicole A., Cooper Stacy, Qayed Muna, Hermiston Michelle, Satwani Prakash, Krupski Christa, Keating Amy, Fabrizio Vanessa, Chinnabhandar Vasant, Kunicki Michael, Curran Kevin J., Mackall Crystal L., Laetsch Theodore W., Schultz Liora M. (2023). Tisagenlecleucel utilisation and outcomes across refractory, first relapse and multiply relapsed B-cell acute lymphoblastic leukemia: a retrospective analysis of real-world patterns. eClinicalMedicine.

[ref-288397] Winestone Lena E., Bhojwani Deepa, Ghorashian Sara, Muffly Lori, Leahy Allison Barz, Chao Karen, Steineck Angela, Rössig Claudia, Lamble Adam, Maude Shannon L., Myers Regina, Rheingold Susan R. (2024). INSPIRED Symposium Part 4A: Access to CAR T Cell Therapy in Unique Populations with B Cell Acute Lymphoblastic Leukemia. Transplantation and Cellular Therapy.

[ref-288398] Leahy Allison Barz, Newman Haley, Li Yimei, Liu Hongyan, Myers Regina, DiNofia Amanda, Dolan Joseph G, Callahan Colleen, Baniewicz Diane, Devine Kaitlin, Wray Lisa, Aplenc Richard, June Carl H, Grupp Stephan A, Rheingold Susan R, Maude Shannon L (2021). CD19-targeted chimeric antigen receptor T-cell therapy for CNS relapsed or refractory acute lymphocytic leukaemia: a post-hoc analysis of pooled data from five clinical trials. The Lancet Haematology.

[ref-288399] Qi Yuekun, Zhao Mingfeng, Hu Yongxian, Wang Ying, Li Ping, Cao Jiang, Shi Ming, Tan Jiaqi, Zhang Meng, Xiao Xia, Xia Jieyun, Ma Sha, Qiao Jianlin, Yan Zhiling, Li Hujun, Pan Bin, Sang Wei, Li Depeng, Li Zhenyu, Zhou Jianfeng, Huang He, Liang Aibin, Zheng Junnian, Xu Kailin (2022). Efficacy and safety of CD19-specific CAR T cell–based therapy in B-cell acute lymphoblastic leukemia patients with CNSL. Blood.

[ref-288400] Fabrizio Vanessa A., Phillips Christine L., Lane Adam, Baggott Christina, Prabhu Snehit, Egeler Emily, Mavroukakis Sharon, Pacenta Holly, Rossoff Jenna, Stefanski Heather E., Talano Julie-An, Moskop Amy, Margossian Steven P., Verneris Michael R., Myers Gary Douglas, Karras Nicole A., Brown Patrick A., Qayed Muna, Hermiston Michelle, Satwani Prakash, Krupski Christa, Keating Amy K., Wilcox Rachel, Rabik Cara A., Chinnabhandar Vasant, Kunicki Michael, Goksenin A. Yasemin, Curran Kevin J., Mackall Crystal L., Laetsch Theodore W., Schultz Liora M. (2022). Tisagenlecleucel outcomes in relapsed/refractory extramedullary ALL: a Pediatric Real World CAR Consortium Report. Blood Adv.

[ref-288401] Jacoby Elad, Ghorashian Sara, Vormoor Britta, De Moerloose Barbara, Bodmer Nicole, Molostova Olga, Yanir Asaf D, Buechner Jochen, Elhasid Ronit, Bielorai Bella, Rogosic Srdan, Dourthe Marie-Emilie, Maschan Michael, Rossig Claudia, Toren Amos, von Stackelberg Arend, Locatelli Franco, Bader Peter, Zimmermann Martin, Bourquin Jean Pierre, Baruchel Andre (2022). CD19 CAR T-cells for pediatric relapsed acute lymphoblastic leukemia with active CNS involvement: a retrospective international study. Leukemia.

[ref-288402] Ghorashian Sara, Jacoby Elad, De Moerloose Barbara, Rives Susana, Bonney Denise, Shenton Geoff, Bader Peter, Bodmer Nicole, Quintana Agueda Molinos, Herrero Blanca, Algeri Mattia, Locatelli Franco, Vettenranta Kim, Gonzalez Berta, Attarbaschi Andishe, Harris Stephen, Bourquin Jean Pierre, Baruchel André (2022). Tisagenlecleucel therapy for relapsed or refractory B-cell acute lymphoblastic leukaemia in infants and children younger than 3 years of age at screening: an international, multicentre, retrospective cohort study. The Lancet Haematology.

[ref-288403] Moskop Amy, Pommert Lauren, Baggott Christina, Prabhu Snehit, Pacenta Holly L., Phillips Christine L., Rossoff Jenna, Stefanski Heather E., Talano Julie-An, Margossian Steve P., Verneris Michael R., Myers G. Doug, Karras Nicole A., Brown Patrick A., Qayed Muna, Hermiston Michelle L., Satwani Prakash, Krupski Christa, Keating Amy K., Wilcox Rachel, Rabik Cara A., Fabrizio Vanessa A., Chinnabhandar Vasant, Goksenin A. Yasemin, Curran Kevin J., Mackall Crystal L., Laetsch Theodore W., Guest Erin M., Breese Erin H., Schultz Liora M. (2022). Real-world use of tisagenlecleucel in infant acute lymphoblastic leukemia. Blood Adv.

[ref-288404] Hitzler Johann K., He Wensheng, Doyle John, Cairo Mitchell, Camitta Bruce M., Chan Ka Wah, Perez Miguel A. Diaz, Fraser Christopher, Gross Thomas G., Horan John T., Kennedy-Nasser Alana A., Kitko Carrie, Kurtzberg Joanne, Lehmann Leslie, O'Brien Tracey, Pulsipher Michael A., Smith Franklin O., Zhang Mei-Jie, Eapen Mary, Carpenter Paul A., On behalf of the CIBMTR Pediatric Cancer Working Committee (2014). Outcome of transplantation for acute lymphoblastic leukemia in children with down syndrome. Pediatric Blood & Cancer.

[ref-288405] Laetsch Theodore W., Maude Shannon L., Balduzzi Adriana, Rives Susana, Bittencourt Henrique, Boyer Michael W., Buechner Jochen, De Moerloose Barbara, Qayed Muna, Phillips Christine L., Pulsipher Michael A., Hiramatsu Hidefumi, Tiwari Ranjan, Grupp Stephan A. (2022). Tisagenlecleucel in pediatric and young adult patients with Down syndrome-associated relapsed/refractory acute lymphoblastic leukemia. Leukemia.

[ref-288406] Qayed Muna, McGuirk Joseph P., Myers G. Doug, Parameswaran Vinod, Waller Edmund K., Holman Peter, Rodrigues Margarida, Clough Lee F., Willert Jennifer (2022). Leukapheresis guidance and best practices for optimal chimeric antigen receptor T-cell manufacturing. Cytotherapy.

[ref-288407] Livingston Joel, Di-Mola Maria, Lowry Jane, Ruse Nigel, Chiang Kuang-Yueh, Chopra Yogi, Schechter Tal, Ali Muhammad, Licht Christoph, Wall Donna, Krueger Joerg (2021). Peripheral venous catheter collection of immune effector cells and hematopoietic stem cells is feasible and safe in older pediatric patients. Transfusion.

[ref-288408] Hutt Daphna, Bielorai Bella, Baturov Bella, Z’orbinski Inna, Ilin Natalia, Adam Etai, Itzhaki Orit, Besser Michal J., Toren Amos, Jacoby Elad (2020). Feasibility of leukapheresis for CAR T-cell production in heavily pre-treated pediatric patients. Transfusion and Apheresis Science.

[ref-288409] Ceppi Francesco, Rivers Julie, Annesley Colleen, Pinto Navin, Park Julie R., Lindgren Catherine, Mgebroff Stephanie, Linn Naomi, Delaney Meghan, Gardner Rebecca A. (2018). Lymphocyte apheresis for chimeric antigen receptor T-cell manufacturing in children and young adults with leukemia and neuroblastoma. Transfusion.

[ref-288410] O'Reilly Maeve A., Malhi Aman, Cheok Kathleen P.L., Ings Stuart, Balsa Carmen, Keane Helen, Jalowiec Katarzyna, Neill Lorna, Peggs Karl S., Roddie Claire (2023). A novel predictive algorithm to personalize autologous T-cell harvest for chimeric antigen receptor T-cell manufacture. Cytotherapy.

[ref-288411] Singh Nathan, Perazzelli Jessica, Grupp Stephan A., Barrett David M. (2016). Early memory phenotypes drive T cell proliferation in patients with pediatric malignancies. Science Translational Medicine.

[ref-288412] Qin Haiying, Ishii Kazusa, Nguyen Sang, Su Paul P., Burk Chad R., Kim Bong-Hyun, Duncan Brynn B., Tarun Samikasha, Shah Nirali N., Kohler M. Eric, Fry Terry J. (2018). Murine pre–B-cell ALL induces T-cell dysfunction not fully reversed by introduction of a chimeric antigen receptor. Blood.

[ref-288413] Perez Perez Ariel, Bachmeier Christina A, Smilee Renee, Ribickas Albert J, Lazaryan Aleksandr, Chavez Julio C., Anasetti Claudio, Shah Bijal, Khimani Farhad, Nishihori Taiga, Davila Marco L., Locke Frederick L., Jain Michael D., Liu Hien D. (2020). Factors Affecting Lymphocyte Collection Efficiency and Manufactured Product Specification during Leukapheresis for Diffuse Large B Cell Lymphoma Patients Treated with Commercial Tisagenlecleucel. Blood.

[ref-288414] Mahadeo Kris M., Khazal Sajad J., Abdel-Azim Hisham, Fitzgerald Julie C., Taraseviciute Agne, Bollard Catherine M., Tewari Priti, Duncan Christine, Traube Chani, McCall David, Steiner Marie E., Cheifetz Ira M., Lehmann Leslie E., Mejia Rodrigo, Slopis John M., Bajwa Rajinder, Kebriaei Partow, Martin Paul L., Moffet Jerelyn, McArthur Jennifer, Petropoulos Demetrios, O’Hanlon Curry Joan, Featherston Sarah, Foglesong Jessica, Shoberu Basirat, Gulbis Alison, Mireles Maria E., Hafemeister Lisa, Nguyen Cathy, Kapoor Neena, Rezvani Katayoun, Neelapu Sattva S., Shpall Elizabeth J., the Pediatric Acute Lung Injury and Sepsis Investigators (PALISI) Network (2018). Management guidelines for paediatric patients receiving chimeric antigen receptor T cell therapy. Nature Reviews Clinical Oncology.

[ref-288415] Turtle Cameron J, Hanafi Laila-Aicha, Berger Carolina, Sommermeyer Daniel, Pender Barbara, Robinson Emily M, Melville Katherine, Budiarto Tanya M, Steevens Natalia N, Chaney Colette, Cherian Sindhu, Wood Brent L, Soma Lorinda, Chen Xueyan, Heimfeld Shelly, Jensen Michael C, Riddell Stanley R., Maloney David G (2015). Addition of Fludarabine to Cyclophosphamide Lymphodepletion Improves In Vivo Expansion of CD19 Chimeric Antigen Receptor-Modified T Cells and Clinical Outcome in Adults with B Cell Acute Lymphoblastic Leukemia. Blood.

[ref-288416] Langenhorst Jurgen B., Dorlo Thomas P. C., van Maarseveen Erik M., Nierkens Stefan, Kuball Jürgen, Boelens Jaap Jan, van Kesteren Charlotte, Huitema Alwin D. R. (2018). Population Pharmacokinetics of Fludarabine in Children and Adults during Conditioning Prior to Allogeneic Hematopoietic Cell Transplantation. Clinical Pharmacokinetics.

[ref-288417] Langenhorst J. B., van Kesteren C., van Maarseveen E. M., Dorlo T. P. C., Nierkens S., Lindemans C. A., de Witte M. A., van Rhenen A., Raijmakers R., Bierings M., Kuball J., Huitema A. D. R., Boelens J. J. (2019). Fludarabine exposure in the conditioning prior to allogeneic hematopoietic cell transplantation predicts outcomes. Blood Adv.

[ref-288418] Dekker Linde, Calkoen Friso G., Jiang Yilin, Blok Hilly, Veldkamp Saskia R., De Koning Coco, Spoon Maike, Admiraal Rick, Hoogerbrugge Peter, Vormoor Britta, Vormoor H. Josef, Visscher Henk, Bierings Marc, Van Der Vlugt Marieke, Van Tinteren Harm, Nijstad A. Laura, Huitema Alwin D. R., Van Der Elst Kim C. M., Pieters Rob, Lindemans Caroline A., Nierkens Stefan (2022). Fludarabine exposure predicts outcome after CD19 CAR T-cell therapy in children and young adults with acute leukemia. Blood Adv.

[ref-288419] Fabrizio Vanessa A., Boelens Jaap Jan, Mauguen Audrey, Baggott Christina, Prabhu Snehit, Egeler Emily, Mavroukakis Sharon, Pacenta Holly, Phillips Christine L., Rossoff Jenna, Stefanski Heather E., Talano Julie-An, Moskop Amy, Margossian Steven P., Verneris Michael R., Myers Gary Douglas, Karras Nicole A., Brown Patrick A., Qayed Muna, Hermiston Michelle, Satwani Prakash, Krupski Christa, Keating Amy K., Wilcox Rachel, Rabik Cara A., Chinnabhandar Vasant, Kunicki Michael, Goksenin A. Yasemin, Mackall Crystal L., Laetsch Theodore W., Schultz Liora M., Curran Kevin J. (2022). Optimal fludarabine lymphodepletion is associated with improved outcomes after CAR T-cell therapy. Blood Adv.

[ref-288420] Bense Joëll E., Haverman Lotte, von Asmuth Erik G.J., Louwerens Marloes, Luijten Michiel A.J., Stiggelbout Anne M., Lankester Arjan C., de Pagter Anne P.J. (2023). Late Effects in Pediatric Allogeneic Hematopoietic Stem Cell Transplantation for Nonmalignant Diseases: Proxy- and Patient-Reported Outcomes. Transplantation and Cellular Therapy.

[ref-288421] Inamoto Yoshihiro, Lee Stephanie J. (2017). Late effects of blood and marrow transplantation. Haematologica.

[ref-288422] Kelly Debra Lynch, Buchbinder David, Duarte Rafael F., Auletta Jeffrey J., Bhatt Neel, Byrne Michael, DeFilipp Zachariah, Gabriel Melissa, Mahindra Anuj, Norkin Maxim, Schoemans Helene, Shah Ami J., Ahmed Ibrahim, Atsuta Yoshiko, Basak Grzegorz W., Beattie Sara, Bhella Sita, Bredeson Christopher, Bunin Nancy, Dalal Jignesh, Daly Andrew, Gajewski James, Gale Robert Peter, Galvin John, Hamadani Mehdi, Hayashi Robert J., Adekola Kehinde, Law Jason, Lee Catherine J., Liesveld Jane, Malone Adriana K., Nagler Arnon, Naik Seema, Nishihori Taiga, Parsons Susan K., Scherwath Angela, Schofield Hannah-Lise, Soiffer Robert, Szer Jeff, Twist Ida, Warwick Anne, Wirk Baldeep M., Yi Jean, Battiwalla Minoo, Flowers Mary E., Savani Bipin, Shaw Bronwen E. (2018). Neurocognitive Dysfunction in Hematopoietic Cell Transplant Recipients: Expert Review from the Late Effects and Quality of Life Working Committee of the Center for International Blood and Marrow Transplant Research and Complications and Quality of Life Working Party of the European Society for Blood and Marrow Transplantation. Biology of Blood and Marrow Transplantation.

[ref-288423] Chow Eric J., Anderson Lynnette, Baker K. Scott, Bhatia Smita, Guilcher Gregory M.T., Huang Jennifer T., Pelletier Wendy, Perkins Joanna L., Rivard Linda S., Schechter Tal, Shah Ami J., Wilson Karla D., Wong Kenneth, Grewal Satkiran S., Armenian Saro H., Meacham Lillian R., Mulrooney Daniel A., Castellino Sharon M. (2016). Late Effects Surveillance Recommendations among Survivors of Childhood Hematopoietic Cell Transplantation: A Children's Oncology Group Report. Biology of Blood and Marrow Transplantation.

[ref-288424] Laetsch Theodore W, Myers Gary Douglas, Baruchel André, Dietz Andrew C, Pulsipher Michael A, Bittencourt Henrique, Buechner Jochen, De Moerloose Barbara, Davis Kara L, Nemecek Eneida, Driscoll Timothy, Mechinaud Francoise, Boissel Nicolas, Rives Susana, Bader Peter, Peters Christina, Sabnis Himalee S, Grupp Stephan A, Yanik Gregory A, Hiramatsu Hidefumi, Stefanski Heather E, Rasouliyan Lawrence, Yi Lan, Shah Sweta, Zhang Jie, Harris Andrew C (2019). Patient-reported quality of life after tisagenlecleucel infusion in children and young adults with relapsed or refractory B-cell acute lymphoblastic leukaemia: a global, single-arm, phase 2 trial. The Lancet Oncology.

[ref-288425] Ruark Julia, Mullane Erin, Cleary Nancy, Cordeiro Ana, Bezerra Evandro D., Wu Vicky, Voutsinas Jenna, Shaw Bronwen E., Flynn Kathryn E., Lee Stephanie J., Turtle Cameron J., Maloney David G., Fann Jesse R., Bar Merav (2020). Patient-Reported Neuropsychiatric Outcomes of Long-Term Survivors after Chimeric Antigen Receptor T Cell Therapy. Biology of Blood and Marrow Transplantation.

[ref-288426] Hoogland Aasha I., Barata Anna, Logue Jennifer, Kommalapati Anuhya, Hyland Kelly A., Nelson Ashley M., Eisel Sarah L., Small Brent J., James Brian W., Christy Shannon M., Bulls Hailey W., Booth-Jones Margaret, Jayani Reena V., Jain Michael D., Mokhtari Sepideh, Chavez Julio C., Lazaryan Aleksandr, Shah Bijal D., Locke Frederick L., Jim Heather S.L. (2022). Change in Neurocognitive Performance Among Patients with Non-Hodgkin Lymphoma in the First Year after Chimeric Antigen Receptor T Cell Therapy. Transplantation and Cellular Therapy.

[ref-288427] Schofield Hannah-Lise T., Fabrizio Vanessa A., Braniecki Suzanne, Pelletier Wendy, Eissa Hesham, Murphy Beverly, Chewning Joseph, Barton Karen D., Embry Leanne M., Levine John E., Schultz Kirk R., Page Kristin M. (2022). Monitoring Neurocognitive Functioning After Pediatric Cellular Therapy or Hematopoietic Cell Transplant: Guidelines From the COG Neurocognition in Cellular Therapies Task Force. Transplantation and cellular therapy.

[ref-288428] Zhou Shujie, Liu Mingguo, Ren Fei, Meng Xiangjiao, Yu Jinming (2021). The landscape of bispecific T cell engager in cancer treatment. Biomarker Research.

[ref-288429] Novartis Pharmaceuticals (2020). Tisagenlecleucel Versus Blinatumomab or Inotuzumab for Adult Patients With Relapsed/Refractory B-cell Precursor Acute Lymphoblastic Leukemia: A Randomized Open Label, Multicenter, Phase III Trial.

[ref-288430] Verneris Michael R., Ma Qiufei, Zhang Jie, Keating Amy, Tiwari Ranjan, Li Junlong, Yang Hongbo, Agarwal Abhijit, Pacaud Lida (2021). Indirect comparison of tisagenlecleucel and blinatumomab in pediatric relapsed/refractory acute lymphoblastic leukemia. Blood Adv.

[ref-288431] Gore Lia, Locatelli Franco, Zugmaier Gerhard, Handgretinger Rupert, O’Brien Maureen M., Bader Peter, Bhojwani Deepa, Schlegel Paul-Gerhardt, Tuglus Catherine A., von Stackelberg Arend (2018). Survival after blinatumomab treatment in pediatric patients with relapsed/refractory B-cell precursor acute lymphoblastic leukemia. Blood Cancer Journal.

[ref-288432] von Stackelberg Arend, Locatelli Franco, Zugmaier Gerhard, Handgretinger Rupert, Trippett Tanya M., Rizzari Carmelo, Bader Peter, O’Brien Maureen M., Brethon Benoît, Bhojwani Deepa, Schlegel Paul Gerhardt, Borkhardt Arndt, Rheingold Susan R., Cooper Todd Michael, Zwaan Christian M., Barnette Phillip, Messina Chiara, Michel Gérard, DuBois Steven G., Hu Kuolung, Zhu Min, Whitlock James A., Gore Lia (2016). Phase I/Phase II Study of Blinatumomab in Pediatric Patients With Relapsed/Refractory Acute Lymphoblastic Leukemia. Journal of Clinical Oncology.

[ref-288433] Hogan Laura E., Brown Patrick A., Ji Lingyun, Xu Xinxin, Devidas Meenakshi, Bhatla Teena, Borowitz Michael J., Raetz Elizabeth A., Carroll Andrew, Heerema Nyla A., Zugmaier Gerhard, Sharon Elad, Bernhardt Melanie B., Terezakis Stephanie A., Gore Lia, Whitlock James A., Hunger Stephen P., Loh Mignon L. (2023). Children's Oncology Group AALL1331: Phase III Trial of Blinatumomab in Children, Adolescents, and Young Adults With Low-Risk B-Cell ALL in First Relapse. Journal of Clinical Oncology.

[ref-288435] Molina John C., Shah Nirali N. (2021). CAR T cells better than BiTEs. Blood Advances.

[ref-288436] Subklewe Marion (2021). BiTEs better than CAR T cells. Blood Advances.

[ref-288437] Queudeville Manon, Stein Anthony S., Locatelli Franco, Ebinger Martin, Handgretinger Rupert, Gökbuget Nicola, Gore Lia, Zeng Yi, Gokani Priya, Zugmaier Gerhard, Kantarjian Hagop M. (2023). Low leukemia burden improves blinatumomab efficacy in patients with relapsed/refractory B-cell acute lymphoblastic leukemia. Cancer.

[ref-288438] Majzner Robbie G., Rietberg Skyler P., Sotillo Elena, Dong Rui, Vachharajani Vipul T., Labanieh Louai, Myklebust June H., Kadapakkam Meena, Weber Evan W., Tousley Aidan M., Richards Rebecca M., Heitzeneder Sabine, Nguyen Sang M., Wiebking Volker, Theruvath Johanna, Lynn Rachel C., Xu Peng, Dunn Alexander R., Vale Ronald D., Mackall Crystal L. (2020). Tuning the Antigen Density Requirement for CAR T-cell Activity. Cancer Discov.

[ref-288439] Libert Diane, Yuan Constance M., Masih Katherine E., Galera Pallavi, Salem Dalia, Shalabi Haneen, Yates Bonnie, Delbrook Cindy, Shern Jack F., Fry Terry J., Khan Javed, Stetler-Stevenson Maryalice, Shah Nirali N. (2020). Serial evaluation of CD19 surface expression in pediatric B-cell malignancies following CD19-targeted therapy. Leukemia.

[ref-288440] Pillai Vinodh, Muralidharan Kavitha, Meng Wenzhao, Bagashev Asen, Oldridge Derek A., Rosenthal Jaclyn, Van Arnam John, Melenhorst Jos J., Mohan Diwakar, DiNofia Amanda M., Luo Minjie, Cherian Sindhu, Fromm Jonathan R., Wertheim Gerald, Thomas-Tikhonenko Andrei, Paessler Michele, June Carl H., Luning Prak Eline T., Bhoj Vijay G., Grupp Stephan A., Maude Shannon L., Rheingold Susan R. (2019). CAR T-cell therapy is effective for CD19-dim B-lymphoblastic leukemia but is impacted by prior blinatumomab therapy. Blood Adv.

[ref-288441] Mueller Karen Thudium, Waldron Edward, Grupp Stephan A., Levine John E., Laetsch Theodore W., Pulsipher Michael A., Boyer Michael W., August Keith J., Hamilton Jason, Awasthi Rakesh, Stein Andrew M., Sickert Denise, Chakraborty Abhijit, Levine Bruce L., June Carl H., Tomassian Lori, Shah Sweta S., Leung Mimi, Taran Tetiana, Wood Patricia A., Maude Shannon L. (2018). Clinical Pharmacology of Tisagenlecleucel in B-cell Acute Lymphoblastic Leukemia. Clin Cancer Res.

[ref-288442] Awasthi Rakesh, Mueller Karen Thudium, Yanik Gregory A., Tam Constantine Si Lun, Rives Susana, McGuirk Joseph, Boyer Michael W., Jäger Ulrich, Baruchel Andre, Myers Gary Douglas, Borchmann Peter, Jaglowski Samantha Mary, Stefanski Heather E, Bishop Michael Russell, Waldron Edward K, Hamilton Jason, Cota Mariana, Bubuteishvili-Pacaud Lida, Waller Edmund K. (2018). Considerations for tisagenlecleucel dosing rationale.. Journal of Clinical Oncology.

[ref-288443] Dourthe Marie-Emilie, Rabian Florence, Yakouben Karima, Chevillon Florian, Cabannes-Hamy Aurélie, Méchinaud Françoise, Grain Audrey, Chaillou Delphine, Rahal Ilhem, Caillat-Zucman Sophie, Lesprit Emmanuelle, Naudin Jérôme, Roupret-Serzec Julie, Parquet Nathalie, Brignier Anne, Guérin-El Khourouj Valérie, Lainey Elodie, Caye-Eude Aurélie, Cavé Hélène, Clappier Emmanuelle, Mathis Stéphanie, Azoulay Elie, Dalle Jean Hugues, Dhédin Nathalie, Madelaine Isabelle, Larghero Jérôme, Boissel Nicolas, Baruchel André (2021). Determinants of CD19-positive vs CD19-negative relapse after tisagenlecleucel for B-cell acute lymphoblastic leukemia. Leukemia.

[ref-288444] Jacoby Elad, Nguyen Sang M., Fountaine Thomas J., Welp Kathryn, Gryder Berkley, Qin Haiying, Yang Yinmeng, Chien Christopher D., Seif Alix E., Lei Haiyan, Song Young K., Khan Javed, Lee Daniel W., Mackall Crystal L., Gardner Rebecca A., Jensen Michael C., Shern Jack F., Fry Terry J. (2016). CD19 CAR immune pressure induces B-precursor acute lymphoblastic leukaemia lineage switch exposing inherent leukaemic plasticity. Nature Communications.

[ref-288445] Pan Jing, Tan Yue, Deng Biping, Tong Chunrong, Hua Lin, Ling Zhuojun, Song Weiliang, Xu Jinlong, Duan Jiajia, Wang Zelin, Guo Huilin, Yu Xinjian, Chang Alex H., Zheng Qinlong, Feng Xiaoming (2020). Frequent occurrence of CD19-negative relapse after CD19 CAR T and consolidation therapy in 14 TP53-mutated r/r B-ALL children. Leukemia.

[ref-288446] Buechner Jochen, Caruana Ignazio, Künkele Annette, Rives Susana, Vettenranta Kim, Bader Peter, Peters Christina, Baruchel André, Calkoen Friso G. (2022). Chimeric Antigen Receptor T-Cell Therapy in Paediatric B-Cell Precursor Acute Lymphoblastic Leukaemia: Curative Treatment Option or Bridge to Transplant?. Frontiers in Pediatrics.

[ref-288447] Pulsipher Michael A., Han Xia, Maude Shannon L., Laetsch Theodore W., Qayed Muna, Rives Susana, Boyer Michael W., Hiramatsu Hidefumi, Yanik Gregory A., Driscoll Tim, Myers G. Doug, Bader Peter, Baruchel Andre, Buechner Jochen, Stefanski Heather E., Kalfoglou Creton, Nguyen Kevin, Waldron Edward R., Thudium Mueller Karen, Maier Harald J., Kari Gabor, Grupp Stephan A. (2021). Next-Generation Sequencing of Minimal Residual Disease for Predicting Relapse after Tisagenlecleucel in Children and Young Adults with Acute Lymphoblastic Leukemia. Blood Cancer Discov.

[ref-288448] Benjamin Reuben, Graham Charlotte, Yallop Deborah, Jozwik Agnieszka, Mirci-Danicar Oana C, Lucchini Giovanna, Pinner Danielle, Jain Nitin, Kantarjian Hagop, Boissel Nicolas, Maus Marcela V, Frigault Matthew J, Baruchel André, Mohty Mohamad, Gianella-Borradori Athos, Binlich Florence, Balandraud Svetlana, Vitry Fabien, Thomas Elisabeth, Philippe Anne, Fouliard Sylvain, Dupouy Sandra, Marchiq Ibtissam, Almena-Carrasco Maria, Ferry Nicolas, Arnould Sylvain, Konto Cyril, Veys Paul, Qasim Waseem, Benjamin Reuben, Graham Charlotte, Yallop Deborah, Jozwik Agnieszka, Pagliuca Antonio, Mufti Ghulam, Patten Piers, Kassam Shireen, Devereux Stephen, Kazmi Majid, Cuthill Kirsty, Potter Victoria, Kuhnl Andrea, Metaxa Victoria, Bonganay Laarni, Stewart Orla, Ellard Rose, Catt Lorraine, Lewis Jen, Farzaneh Farzin, Chappell Jackie, Mason Alice, Chu Vicky, Dunlop Alan, Saleem Adeel, Cheung Gary, Munro Helena, Giemza Elka, Qasim Waseem, Veys Paul, Ciocarlie Oana, Lucchini Giovanna, Pinner Danielle, Chu Jan, Amrolia Persis, Rao Kanchan, Chiesa Robert, Silva Juliana, Hill Annette, Finch Maria, Young Lindsey, Hara Harvinder, Samarasinghe Sujith, Rao Anupama, Vora Ajay, Gilmour Kimberley, Rivat Christine, Murphy Clare, Ahsan Gulrukh, Said Shamsah Rasha, James Jesmina, Inglott Sarah, Wright Gary, Adams Stuart, Izotova Natalia, Jain Nitin, Konopleva Marina, Wierda William, Jabbour Elias, Kantarjian Hagop, Kebrieai Partow, Jones Emily, McGee Kara, Maus Marcela, Frigault Matthew, Brown Jami, Toncheva Vesselina, Casey Keagan, Hock Hanno, McKeown Meaghan A, Mathews Richard, Spitzer Thomas, Boissel Nicolas, Raffoux Emmanuel, Lengliné Etienne, Itzykson Raphael, Rabian Florence, Larghero Jérôme, Madelaine Isabelle, Azoulay Elie, Clappier Emmanuelle, Caillat-Zucman Sophie, Meunier Martine, Celli-Lebras Karine, Tremorin Marie-Thérèse, Baruchel André, Yakouben Karima, Mechinaud-Heloury Françoise, Grain Audrey, Alimi Aurélia, Roupret Julie, Chaillou Delphine, Larghero Jérôme, Madelaine Isabelle, Cavé Hélène, Caye-Eude Aurelie, Fenneteau Odile, Lainey Elodie, Naudin Jerome, Mohty Mohamad, Brissot Eolia, Dulery Remy, Malard Florent, Mediavilla Clémence, Bonnin Agnès, Vekhoff Anne, Ledraa Tounes, Larghero Jérôme, Daguenel-Nguyen Anne (2020). Genome-edited, donor-derived allogeneic anti-CD19 chimeric antigen receptor T cells in paediatric and adult B-cell acute lymphoblastic leukaemia: results of two phase 1 studies. The Lancet.

[ref-288449] Qasim Waseem, Zhan Hong, Samarasinghe Sujith, Adams Stuart, Amrolia Persis, Stafford Sian, Butler Katie, Rivat Christine, Wright Gary, Somana Kathy, Ghorashian Sara, Pinner Danielle, Ahsan Gul, Gilmour Kimberly, Lucchini Giovanna, Inglott Sarah, Mifsud William, Chiesa Robert, Peggs Karl S., Chan Lucas, Farzaneh Farzin, Thrasher Adrian J., Vora Ajay, Pule Martin, Veys Paul (2017). Molecular remission of infant B-ALL after infusion of universal TALEN gene-edited CAR T cells. Science Translational Medicine.

[ref-288451] Ghobadi Armin, Aldoss Ibrahim, Maude Shannon L., Bhojwani Deepa, Wayne Alan S., Bajel Ashish, Faramand Rawan, Mattison Ryan J, Dholaria Bhagirathbhai, Rettig Michael P., Jacobs Ken, Bakkacha Ouiam, Muth John, Pannunzio Angela, Ramsey Brett, McNulty Eileen, Cooper Matt L., Davidson-Moncada Jan, DiPersio John F. (2023). Phase 1/2 Dose-Escalation/Dose-Expansion Study of Anti-CD7 Allogeneic CAR-T Cells (WU-CART-007) in Relapsed or Refractory (R/R) T-Cell Acute Lymphoblastic Leukemia/ Lymphoblastic Lymphoma (T-ALL/LBL). Blood.

[ref-288452] Ghorashian Sara, Kramer Anne Marijn, Onuoha Shimobi, Wright Gary, Bartram Jack, Richardson Rachel, Albon Sarah J., Casanovas-Company Joan, Castro Fernanda, Popova Bilyana, Villanueva Krystle, Yeung Jenny, Vetharoy Winston, Guvenel Aleks, Wawrzyniecka Patrycja A., Mekkaoui Leila, Cheung Gordon Weng-Kit, Pinner Danielle, Chu Jan, Lucchini Giovanna, Silva Juliana, Ciocarlie Oana, Lazareva Arina, Inglott Sarah, Gilmour Kimberly C., Ahsan Gulrukh, Ferrari Mathieu, Manzoor Somayya, Champion Kim, Brooks Tony, Lopes Andre, Hackshaw Allan, Farzaneh Farzin, Chiesa Robert, Rao Kanchan, Bonney Denise, Samarasinghe Sujith, Goulden Nicholas, Vora Ajay, Veys Paul, Hough Rachael, Wynn Robert, Pule Martin A., Amrolia Persis J. (2019). Enhanced CAR T cell expansion and prolonged persistence in pediatric patients with ALL treated with a low-affinity CD19 CAR. Nature Medicine.

[ref-288453] Fry Terry J, Shah Nirali N, Orentas Rimas J, Stetler-Stevenson Maryalice, Yuan Constance M, Ramakrishna Sneha, Wolters Pamela, Martin Staci, Delbrook Cindy, Yates Bonnie, Shalabi Haneen, Fountaine Thomas J, Shern Jack F, Majzner Robbie G, Stroncek David F, Sabatino Marianna, Feng Yang, Dimitrov Dimiter S, Zhang Ling, Nguyen Sang, Qin Haiying, Dropulic Boro, Lee Daniel W, Mackall Crystal L (2017). CD22-targeted CAR T cells induce remission in B-ALL that is naive or resistant to CD19-targeted CAR immunotherapy. Nature medicine.

[ref-288454] Cordoba Shaun, Onuoha Shimobi, Thomas Simon, Pignataro Daniela Soriano, Hough Rachael, Ghorashian Sara, Vora Ajay, Bonney Denise, Veys Paul, Rao Kanchan, Lucchini Giovanna, Chiesa Robert, Chu Jan, Clark Liz, Fung Mei Mei, Smith Koval, Peticone Carlotta, Al-Hajj Muhammad, Baldan Vania, Ferrari Mathieu, Srivastava Saket, Jha Ram, Arce Vargas Frederick, Duffy Kevin, Day William, Virgo Paul, Wheeler Lucy, Hancock Jeremy, Farzaneh Farzin, Domning Sabine, Zhang Yiyun, Khokhar Nushmia Z., Peddareddigari Vijay G. R., Wynn Robert, Pule Martin, Amrolia Persis J. (2021). CAR T cells with dual targeting of CD19 and CD22 in pediatric and young adult patients with relapsed or refractory B cell acute lymphoblastic leukemia: a phase 1 trial. Nature Medicine.

[ref-288455] Shalabi Haneen, Qin Haiying, Su Angela, Yates Bonnie, Wolters Pamela L., Steinberg Seth M., Ligon John A., Silbert Sara, DéDé Kniya, Benzaoui Mehdi, Goldberg Sophia, Achar Sooraj, Schneider Dina, Shahani Shilpa A., Little Lauren, Foley Toni, Molina John C., Panch Sandhya, Mackall Crystal L., Lee Daniel W., Chien Christopher D., Pouzolles Marie, Ahlman Mark, Yuan Constance M., Wang Hao-Wei, Wang Yanyu, Inglefield Jon, Toledo-Tamula Mary Anne, Martin Staci, Highfill Steven L., Altan-Bonnet Gregoire, Stroncek David, Fry Terry J., Taylor Naomi, Shah Nirali N. (2022). CD19/22 CAR T cells in children and young adults with B-ALL: phase 1 results and development of a novel bicistronic CAR. Blood.

[ref-288456] Novartis five-year Kymriah® data show durable remission and long-term survival maintained in children and young adults with advanced B-cell ALL.

[ref-288457] Oluwole Olalekan O., Dholaria Bhagirathbhai, Knight Tristan E., Jain Tania, Locke Frederick L., Ramsdell Linda, Nikiforow Sarah, Hashmi Hamza, Mooney Kathy, Bhaskar Shakthi T., Morris Katrina, Gatwood Katie, Baer Brittney, Anderson Larry D. Jr., Hamadani Mehdi (2024). Chimeric Antigen Receptor T-Cell Therapy in the Outpatient Setting: An Expert Panel Opinion from the American Society for Transplantation and Cellular Therapy. Transplantation and Cellular Therapy.

[ref-288458] Bansal Radhika, Paludo Jonas, Holland Adam, Megan Spychalla, Alli McClanahan, Hathcock Matthew, Alkhateeb Hassan, Dingli David, Wang Yucai, Kenderian Saad, Kumar Shaji, Shah Mithun Vinod, Mustaqeem Siddiqui, Warsame Rahma M., Villasboas Jose Caetano, Bennani Nabila Nora, Johnston Patrick B., Ansell Stephen M., Haddad Tufia C., Lin Yi (2021). Outpatient practice pattern and remote patient monitoring for axicabtagene ciloleucel CAR-T therapy in patients with aggressive lymphoma.. Journal of Clinical Oncology.

[ref-288459] Nasta Sunita D., Hughes Mitchell E., Namoglu Esin C., Garfall Alfred, DiFilippo Heather, Ballard Hatcher J., Barta Stefan K., Chong Elise A., Frey Noelle V., Gerson James N., Landsburg Daniel J., Ruella Marco, Schuster Stephen J., Svoboda Jakub, Weber Elizabeth, Porter David L. (2022). Outcomes of Tisagenlecleucel in Lymphoma Patients With Predominant Management in an Ambulatory Setting. Clinical Lymphoma Myeloma and Leukemia.

[ref-288460] McGann Mary, Davis James A., Gaffney Kelly J., Smith Deidra, Edwards Kathy, Hess Brian T., Hashmi Hamza (2022). Real-World Experience and Optimization of Outpatient Chimeric Antigen Receptor T Cell Therapy. Transplantation and Cellular Therapy.

[ref-288461] Dholaria Bhagirathbhai, Mehraban Nasima, Baer Brittney, Long Nancy, Jayani Reena V., Byrne Michael T., Kassim Adetola A., Engelhardt Brian G., Savani Bipin N., Oluwole Olalekan O. (2022). Feasibility of outpatient administration of axicabtagene ciloleucel and brexucabtagene autoleucel using telemedicine tools: The Vanderbilt experience. British Journal of Haematology.

[ref-288462] Ahmed Nausheen, Wesson William, Mushtaq Muhammad Umair, Porter David L., Nasta Sunita D., Brower Jamie, Bachanova Veronika, Hu Marie, Nastoupil Loretta J., Oluwole Olalekan O., Patel Vivek G., Oliai Caspian, Riedell Peter A., Bishop Michael R., Shah Gunjan L., Perales Miguel-Angel, Schachter Levanto, Maziarz Richard T., McGuirk Joseph P. (2023). Patient Characteristics and Outcomes of Outpatient Tisagenlecleucel Recipients for B Cell Non-Hodgkin Lymphoma. Transplantation and Cellular Therapy.

[ref-288463] Ly Andrew, Sanber Khaled, Tsai Hua-Ling, Ondo Angela, Mooney Kathy, Shedeck Audra, Baker Julie, Imus Philip Hollingsworth, Wagner-Johnston Nina, Jain Tania (2023). Outpatient CD19-directed CAR T-cell therapy is feasible in patients of all ages. British Journal of Haematology.

[ref-288464] Borogovac Azra, Keruakous Amany, Bycko Michelle, Holter Chakrabarty Jennifer, Ibrahimi Sami, Khawandanah Mohamad, Selby George B., Yuen Carrie, Schmidt Sarah, Autry Marcus T., Al-Juhaishi Taha, Wieduwilt Matthew J., Asch Adam S. (2022). Safety and feasibility of outpatient chimeric antigen receptor (CAR) T-cell therapy: experience from a tertiary care center. Bone Marrow Transplantation.

[ref-288465] Myers G Doug, Verneris Michael R, Goy Andre, Maziarz Richard T (2021). Perspectives on outpatient administration of CAR-T cell therapy in aggressive B-cell lymphoma and acute lymphoblastic leukemia. Journal for ImmunoTherapy of Cancer.

[ref-288466] Borogovac Azra, Keruakous Amany R., Bycko Michelle, Holter Chakrabarty Jennifer, Ibrahimi Sami, Khawandanah Mohamad O., Selby George B., Yuen Carrie, Schmidt Sarah A., Al-Juhaishi Taha, Wieduwilt Matthew, Asch Adam S. (2021). Successful Development of an Outpatient Chimeric Antigen Receptor (CAR) T Cell Therapy Program. Blood.

[ref-288467] Zarychta Julia, Kowalczyk Adrian, Krawczyk Milena, Lejman Monika, Zawitkowska Joanna (2023). CAR-T Cells Immunotherapies for the Treatment of Acute Myeloid Leukemia—Recent Advances. Cancers.

[ref-288468] Molica Matteo, Perrone Salvatore, Mazzone Carla, Niscola Pasquale, Cesini Laura, Abruzzese Elisabetta, de Fabritiis Paolo (2021). CD33 Expression and Gentuzumab Ozogamicin in Acute Myeloid Leukemia: Two Sides of the Same Coin. Cancers.

[ref-288469] El Achi Hanadi, Dupont Edouard, Paul Shilpa, Khoury Joseph D. (2020). CD123 as a Biomarker in Hematolymphoid Malignancies: Principles of Detection and Targeted Therapies. Cancers.

[ref-288470] Cummins Katherine, Gill Saar (2023). Chimeric Antigen Receptor T Cells in Acute Myeloid Leukemia. Hematology/Oncology Clinics of North America.

[ref-288471] Pei Kunlin, Xu Haoyu, Wang Pengfei, Gan Wening, Hu Zhengbin, Su Xiaoling, Zhang Hui, He Yingyi (2023). Anti-CLL1-based CAR T-cells with 4-1-BB or CD28/CD27 stimulatory domains in treating childhood refractory/relapsed acute myeloid leukemia. Cancer Medicine.

[ref-288472] Zhang Hui, Bu Chaoke, Peng Zhiyong, Li Guangchao, Zhou Zhao, Ding Wen, Zheng Yongwei, He Yingyi, Hu Zhengbin, Pei Kunlin, Luo Min, Li Chunfu (2022). Characteristics of anti-CLL1 based CAR-T therapy for children with relapsed or refractory acute myeloid leukemia: the multi-center efficacy and safety interim analysis. Leukemia.

[ref-288473] Del Bufalo Francesca, De Angelis Biagio, Caruana Ignazio, Del Baldo Giada, De Ioris Maria A., Serra Annalisa, Mastronuzzi Angela, Cefalo Maria G., Pagliara Daria, Amicucci Matteo, Li Pira Giuseppina, Leone Giovanna, Bertaina Valentina, Sinibaldi Matilde, Di Cecca Stefano, Guercio Marika, Abbaszadeh Zeinab, Iaffaldano Laura, Gunetti Monica, Iacovelli Stefano, Bugianesi Rossana, Macchia Stefania, Algeri Mattia, Merli Pietro, Galaverna Federica, Abbas Rachid, Garganese Maria C., Villani Maria F., Colafati Giovanna S., Bonetti Federico, Rabusin Marco, Perruccio Katia, Folsi Veronica, Quintarelli Concetta, Locatelli Franco (2023). GD2-CART01 for Relapsed or Refractory High-Risk Neuroblastoma. New England Journal of Medicine.

[ref-288474] Del Baldo Giada, Del Bufalo Francesca, Pinacchio Claudia, Carai Andrea, Quintarelli Concetta, De Angelis Biagio, Merli Pietro, Cacchione Antonella, Locatelli Franco, Mastronuzzi Angela (2023). The peculiar challenge of bringing CAR-T cells into the brain: Perspectives in the clinical application to the treatment of pediatric central nervous system tumors. Frontiers in Immunology.

[ref-288475] Guzman Grace, Pellot Karolina, Reed Megan R., Rodriguez Analiz (2023). CAR T-cells to treat brain tumors. Brain Research Bulletin.

[ref-288476] Desland Fiona A., Hormigo Adília (2020). The CNS and the Brain Tumor Microenvironment: Implications for Glioblastoma Immunotherapy. International Journal of Molecular Sciences.

[ref-288477] Kankeu Fonkoua Lionel A., Sirpilla Olivia, Sakemura Reona, Siegler Elizabeth L., Kenderian Saad S. (2022). CAR T cell therapy and the tumor microenvironment: Current challenges and opportunities. Molecular Therapy - Oncolytics.

[ref-288478] Thomas Pauline, Galopin Natacha, Bonérandi Emma, Clémenceau Béatrice, Fougeray Sophie, Birklé Stéphane (2021). CAR T Cell Therapy’s Potential for Pediatric Brain Tumors. Cancers.

[ref-288479] Vitanza Nicholas A., Wilson Ashley L., Huang Wenjun, Seidel Kristy, Brown Christopher, Gustafson Joshua A., Yokoyama Jason K., Johnson Adam J., Baxter Blake A., Koning Ryan W., Reid Aquene N., Meechan Michael, Biery Matthew C., Myers Carrie, Rawlings-Rhea Stephanie D., Albert Catherine M., Browd Samuel R., Hauptman Jason S., Lee Amy, Ojemann Jeffrey G., Berens Michael E., Dun Matthew D., Foster Jessica B., Crotty Erin E., Leary Sarah E.S., Cole Bonnie L., Perez Francisco A., Wright Jason N., Orentas Rimas J., Chour Tony, Newell Evan W., Whiteaker Jeffrey R., Zhao Lei, Paulovich Amanda G., Pinto Navin, Gust Juliane, Gardner Rebecca A., Jensen Michael C., Park Julie R. (2022). Intraventricular B7-H3 CAR T Cells for Diffuse Intrinsic Pontine Glioma: Preliminary First-in-Human Bioactivity and Safety. Cancer Discov.

[ref-288480] Vitanza Nicholas A., Johnson Adam J., Wilson Ashley L., Brown Christopher, Yokoyama Jason K., Künkele Annette, Chang Cindy A., Rawlings-Rhea Stephanie, Huang Wenjun, Seidel Kristy, Albert Catherine M., Pinto Navin, Gust Juliane, Finn Laura S., Ojemann Jeffrey G., Wright Jason, Orentas Rimas J., Baldwin Michael, Gardner Rebecca A., Jensen Michael C., Park Julie R. (2021). Locoregional infusion of HER2-specific CAR T cells in children and young adults with recurrent or refractory CNS tumors: an interim analysis. Nature Medicine.

[ref-288481] Ravanpay Ali C., Gust Juliane, Johnson Adam J., Rolczynski Lisa S., Cecchini Michelle, Chang Cindy A., Hoglund Virginia J., Mukherjee Rithun, Vitanza Nicholas A., Orentas Rimas J., Jensen Michael C. (2019). EGFR806-CAR T cells selectively target a tumor-restricted EGFR epitope in glioblastoma. Oncotarget.

